# Investigation of the impact of COVID‐19 on postoperative outcomes using a nationwide Japanese database of patients undergoing laparoscopic distal gastrectomy and low anterior resection for gastric cancer and rectal cancer

**DOI:** 10.1002/ags3.12776

**Published:** 2024-01-28

**Authors:** Tomonori Akagi, Hideki Endo, Masafumi Inomata, Hidefumi Shiroshita, Shigeki Yamaguchi, Susumu Eguchi, Norihito Wada, Yukinori Kurokawa, Yosuke Seki, Yoshiharu Sakai, Hiroyuki Yamamoto, Yoshihiro Kakeji, Yuko Kitagawa, Akinobu Taketomi, Masaki Mori

**Affiliations:** ^1^ Academic committee of Japan Society for Endoscopic Surgery Tokyo Japan; ^2^ Department of Gastroenterological and Pediatric Surgery Oita University Faculty of Medicine Oita Japan; ^3^ Department of Healthcare Quality Assessment, Graduate School of Medicine The University of Tokyo Tokyo Japan; ^4^ Department of Surgery, Division of Colorectal Surgery Tokyo Women's Medical University Tokyo Japan; ^5^ Department of Surgery Nagasaki University Graduate School of Biomedical Sciences Nagasaki Japan; ^6^ Department of Surgery Shonan Keiiku Hospital Kanagawa Japan; ^7^ Department of Gastroenterological Surgery Osaka University Graduate School of Medicine Osaka Japan; ^8^ Weight Loss and Metabolic Surgery Center Yotsuya Medical Cube Tokyo Japan; ^9^ Japanese Red Cross Osaka Hospital Osaka Japan; ^10^ Division of Gastrointestinal Surgery, Department of Surgery Kobe University Graduate School of Medicine Hyogo Japan; ^11^ Department of Surgery Keio University School of Medicine Tokyo Japan; ^12^ Department of Gastroenterological Surgery I Hokkaido University Graduate School of Medicine Hokkaido Japan; ^13^ President the Japan Surgical Society Tokyo Japan

**Keywords:** COVID‐19, endoscopic surgery, national clinical database

## Abstract

**Background:**

The COVID‐19 outbreak made conventional medical care impossible, forcing changes in both healthcare providers and patients. In Japan, COVID‐19 infection began spreading in earnest in 2020 and exploded in 2021. There was concern that the medical impact of COVID‐19 in 2021 would differ from that in 2020. We aimed to clarify the impact of COVID‐19 on mortality and anastomotic leakage in laparoscopic surgery for gastric cancer and rectal cancer in Japan using the National Clinical Database (NCD).

**Methods:**

We collected data from patients who underwent laparoscopic distal gastrectomy (LDG) and laparoscopic low anterior resection (LLAR) from January 2018 to December 2021 from the NCD, a web‐based surgical registration system in Japan. The number of surgical cases, monthly incidence of mortality and morbidity (anastomotic leakage), standardized mortality ratio (SMR), and standardized morbidity‐leakage ratio (SMLR [ratio of observed patients to expected patients calculated using the risk calculator established in the NCD]) were evaluated.

**Results:**

The numbers of LDG and LLAR cases continued to decline in the first year of the pandemic in 2020 and were as low in 2021 as in 2020. Although the numbers of robot‐assisted LDG and LLAR cases increased, the growth rate was lower than the rate of increase prior to the pandemic. Mortality and anastomotic leakage, two of the most important complications, as assessed by SMR and SMLR, did not worsen during the pandemic in comparison to the pre‐pandemic period.

**Conclusions:**

Laparoscopic surgeries were performed safely in Japan and were not affected by the COVID‐19 pandemic.

## Graphical abstract

Morbidity of LDG and LLAR (anastomotic leakage).

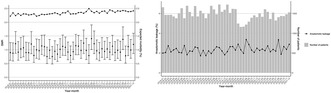

## 1 INTRODUCTION

The first case of infection with the novel coronavirus that was named coronavirus disease 2019 (COVID‐19) by the WHO was first reported in Japan on January 15, 2020.^1^ Since then, the infection has repeatedly spread and disappeared and has not yet been controlled. During this pandemic, government and medical institutions continuously faced two conflicting challenges: treating patients with COVID‐19 without acquiring the infection or causing nosocomial infections and maintaining hospital functions at the same level as before the pandemic,^2^ especially for critically ill patients, such as patients with malignant diseases and acute abdomen. We have previously reported the impact of 2020, the first year that laparoscopic surgery was affected by COVID‐19.^3^ We herein report the impact of COVID‐19 on laparoscopic surgery in 2021, when the infection was more widespread and increased and trends in risk‐adjusted outcomes, including mortality and morbidity, were evaluated.

The pandemic has brought unprecedented changes to both healthcare providers and patients receiving medical care. The possibility of increased mortality in colorectal cancer treatment has been reported from the United Kingdom, but the prevalence of infection differs between countries, as do the conditions of medical care provided in each country.^4^ Furthermore, COVID‐19 spread in earnest in Japan in 2020 and exploded in 2021; there was concern that the medical impact of COVID‐19 in 2021 would be very different than that in 2020.

The Japan Surgical Society guidelines on COVID‐19 warned surgeons to recognize that laparoscopic surgery causes aerosol development and that it should be performed after confirming that the conditions were appropriate (e.g., having a highly precise filter and effluent gas device).^5^ During the COVID‐19 pandemic, medical materials and surgical instruments necessary for laparoscopic surgery were insufficient due to challenges in transportation. According to the JSES survey, the majority of surgical approaches to gastric and colorectal cancer are laparoscopic procedures.^6^ In this study, we evaluated the surgical outcomes of laparoscopic distal gastrectomy (LDG) and laparoscopic low anterior resection (LLAR), which are the main surgical approaches for gastric and colorectal cancer.

In this context, the COVID‐19 pandemic was believed to have an influence on laparoscopic surgeries. In addition, previous studies have confirmed a higher incidence of mortality and pulmonary complications in patients with perioperative COVID‐19 infection.^7^, ^8^, ^9^, ^10^ The purpose of this study was to clarify the impact of the COVID‐19 pandemic on mortality and morbidity in patients undergoing laparoscopic surgery for gastric cancer and colorectal cancer in Japan in 2021 using the National Clinical Database (NCD).

## 2 METHODS

### 2.1 Patients

This study was performed by analyzing essential data extracted from the NCD, a nationwide registry system in Japan that has been linked with the surgical board certification system since 2011. Details on data registration in the Japanese NCD system were described previously.^2^, ^11^ As of 2018, over 5000 institutions had participated in this system, with approximately 1.5 million surgical cases being registered annually. All surgical cases are registered in the NCD, and details, including morbidities, comorbidities, postoperative complications, and mortality, are added to the system.

Distal gastrectomy (DG) and low anterior resection (LAR) were selected, as these are the most common types of laparoscopic surgeries in Japan.^3^ In total, 94 208 cases of DG and 61 869 cases of LAR were registered in the NCD between 2018 and 2021. Among them, patients of <18 years of age and patients who underwent emergent surgeries were excluded from this study. Furthermore, in the DG and LAR groups, cases involving benign disease, malignant disease of organs other than the stomach, and those diseases where the tumor depth (T) or node metastasis (N) were not clear were excluded. Cases with incomplete DG and LAR data were also excluded.

### 2.2 Classification of prefectures according to the degree of infection

The degree of infection in each prefecture was indicated by the cumulative number of infected individuals per population (at the end of 2021). Based on this value, prefectures were classified into the high infection and low infection groups according to the degree of infection. The high infection group included 12 prefectures, namely, Aichi, Chiba, Fukuoka, Hokkaido, Hyogo, Kanagawa, Kyoto, Nara, Okinawa, Osaka, Saitama, and Tokyo. All other prefectures were classified into the low infection group.

### 2.3 Study endpoint

The primary outcome measure of this study was to identify the impact of the COVID‐19 pandemic on operative mortality and morbidity (anastomotic leakage) after laparoscopic surgery of LDG and LLAR. Operative mortality was defined as 30‐day mortality including death after discharge or in‐hospital mortality during the index admission.

### 2.4 Clinical factors

The clinical factors included age at surgery (<65, 65–75, and >75 years); sex (male or female); body mass index (BMI; ≤25 and ≥25 kg/m^2^); smoking history (Brinkman index: 0, <400, and ≥400); presence of preoperative chemotherapy, diabetes mellitus, habitual alcohol intake, chronic obstructive pulmonary disease, congestive heart disease, ischemic heart disease, hypertension, need for preoperative dialysis, previous cerebrovascular disease, chronic steroid use, weight loss, bleeding disorder, and preoperative blood transfusion; American Society of Anesthesiologists physical status (ASA‐PS: 1, 2, and 3–5); and clinical T, N, and M stages. The 7th edition of the American Joint Committee on Cancer TNM classification was used to extract representative T, N, and distant metastasis (M) information. A comparative analysis was conducted between groups in terms of the duration of surgery, intraoperative blood loss, and necessity of transfusion.

A longitudinal graph was visualized using Stata/BE 17 for Mac (StataCorp), and all statistical analyses were performed using R version 4.1.2 (2021; R Foundation for Statistical Computing). Moreover, the standardized mortality and morbidity ratio (SMR) was defined as the ratio of the observed number of patients to the expected number of patients who experienced complications. This ratio was used to investigate the trends in risk‐adjusted outcomes. Expected morbidity and mortality rates for each month were calculated using a risk calculator established by a previous study based on NCD data. The models for operative mortality and anastomotic leakage of DG and LAR were constructed using variables such as sex, age, activities of daily life before surgery, comorbidities, and abnormal hematological findings based on logistic regression analyses.^12^, ^13^, ^14^, ^15^ There is a significant difference between the observed number of patients and the expected number of patients if the 95% confidence interval does not contain 1.

The study protocol was approved, and consent was obtained by the institutional review board of Oita University (approval number: 2444).

## 3 RESULTS

### 3.1 Annual numbers of each procedure (2018–2021)

**FIGURE 1:**
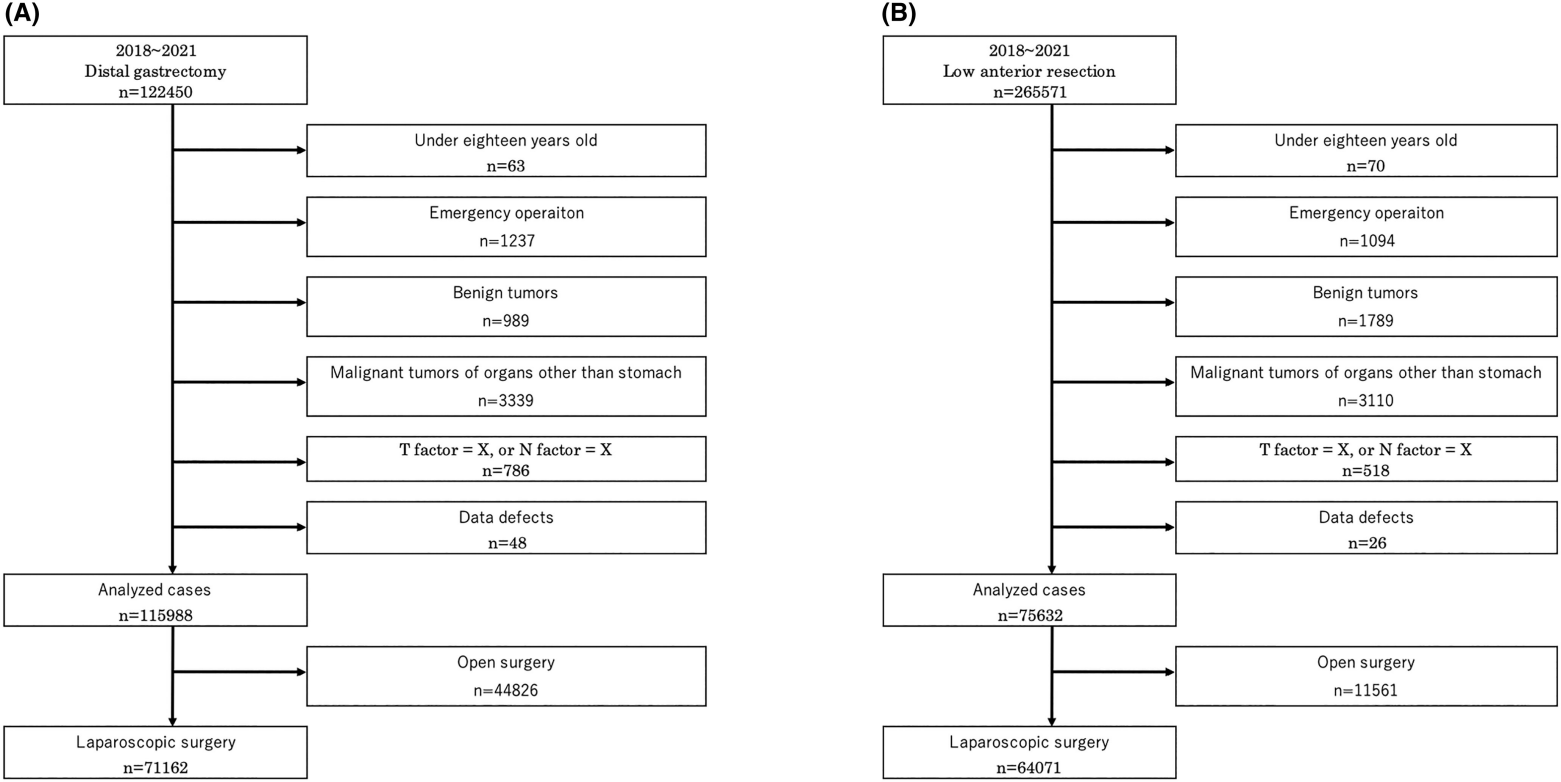
Flowchart detailing the patient selection process. (A) Laparoscopic distal gastrectomy. (B) Laparoscopic low anterior resection.

A flowchart detailing the patient selection process from 2018 to 2021—the study period that was analyzed—is shown in Figure 1, whereas the number of procedures for each year are shown in Table 1. The number of cases treated by each procedure in the two prefectural groups (high and low infection) is shown in Table 2. In the high infection group, for DG, the number of open surgeries (ODGs) in 2021 was 74.3% of that in 2019 and 2020 and 66.2% of that in 2018. The number of LDGs in 2021 was 102.8% of that in 2020 and 85.0% of that in 2019. The number of open low anterior resections (OLARs) in 2021 was 80.9% of that in 2020 and 64.2% of that in 2019. The number of LLARs in 2021 was 97.1% of that in 2020 and 83.4% of that in 2019. The number of robot‐assisted low anterior resections (RLARs) in 2021 was 127.8% of that in 2020 and 181.0% of that in 2019. In the low group, for DG, the number of ODGs in 2021 was 78.1% of that in 2019 and 2020 and 69.9% of that in 2018. The number of LDGs in 2021 was 98.0% of that in 2020 and 88.1% of that in 2019. The number of OLARs in 2021 was 86.0% of that in 2020 and 70.2% of that in 2019. The number of LLARs in 2021 was 98.8% of that in 2019 and 90.5% of that in 2019. The number of RLARs in 2021 was 134.7% of that in 2020 and 189.7% of that in 2019.

**TABLE 1:** Number of surgeries.

	Number of operations (2018)	Number of operations (2019)	Number of operations (2020)	Number of operations (2021)	Vs. 2018	Vs. 2019	Vs. 2020
Distal gastrectomy							
Open	13 531	12 085	10 012	9199	68.0%	76.1%	91.9%
Laparoscopic	16 895	16 610	14 271	14 367	85.0%	86.5%	100.7%
Robot assisted	1159	2102	2553	3213	277.2%	152.9%	125.9%
Low anterior resection							
Open	3732	3159	2544	2126	57.0%	67.3%	83.6%
Laparoscopic	14 308	13 977	12 316	12 077	84.4%	86.4%	98.1%
Robot assisted	1012	2448	3460	4503	445.0%	183.9%	130.1%

**TABLE 2:** Numbers of surgeries according to degree of infection.

	Number of operations (2018)	Number of operations (2019)	Number of operations (2020)	Number of operations (2021)	Vs. 2018	Vs. 2019	Vs. 2020
High infection group							
Distal gastrectomy							
Open	6928	6176	5055	4586	66.2%	74.3%	90.7%
Laparoscopic	9844	9585	7931	8152	82.8%	85.0%	102.8%
Robot assisted	708	1306	1609	1976	279.1%	151.3%	122.8%
Low anterior resection							
Open	1905	1546	1227	993	52.1%	64.2%	80.9%
Laparoscopic	8599	8450	7254	7046	81.9%	83.4%	97.1%
Robot assisted	640	1612	2283	2917	455.8%	181.0%	127.8%
Low infection group							
Distal gastrectomy							
Open	6603	5909	4957	4613	69.9%	78.1%	93.1%
Laparoscopic	7051	7052	6340	6215	88.1%	88.1%	98.0%
Robot assisted	451	796	944	1237	274.3%	155.4%	131.0%
Low anterior resection							
Open	1827	1613	1317	1133	62.0%	70.2%	86.0%
Laparoscopic	5709	5527	5062	5001	87.6%	90.5%	98.8%
Robot assisted	372	836	1177	1586	426.3%	189.7%	134.7%

### 3.2 Trends in the annual ratios of clinicopathological features (2018–2021)

**FIGURE 2:**
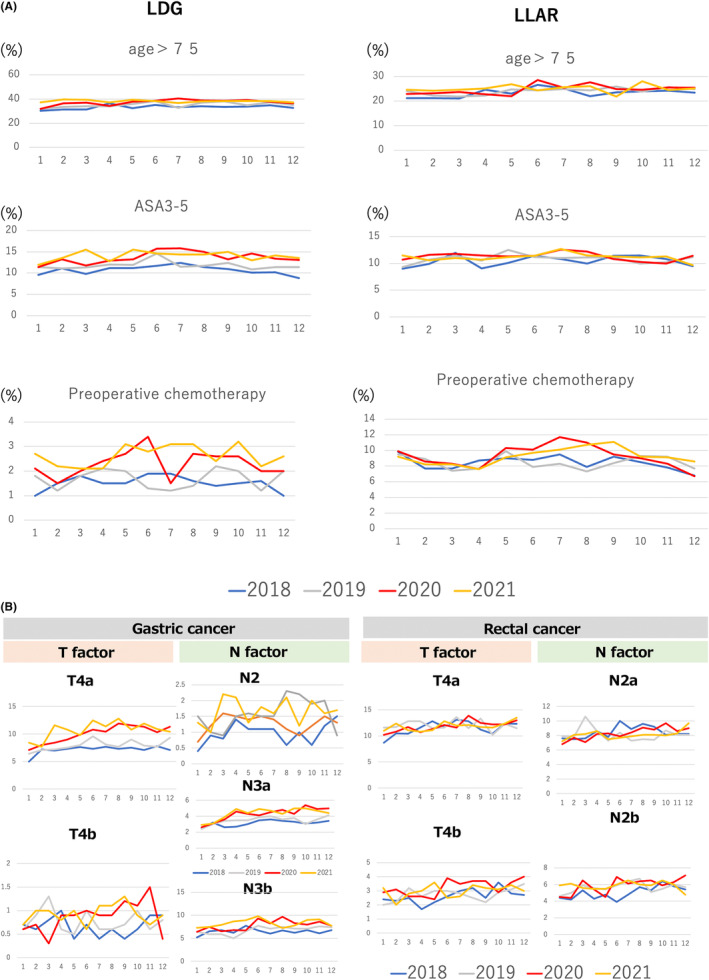
Monthly trend of each procedure. (A) Monthly trends in patient characteristics. (B) Monthly trends in tumor characteristics.

Figure 2A,B shows the monthly trends in patient and tumor characteristics. In 2021, both patients who received LDG and LAR were more likely to have ASA 3–5. We extracted data for the month of August, when the Tokyo Olympics were held, which is feared to have led to the spread of the infection and led to the sixth wave in Japan. The proportion of patients who received LDG with preoperative chemotherapy in August was 1.6% in 2018, 1.4% in 2019, 2.7% in 2020, and 3.1% in 2021. The proportion of T4a patients who received LDG in August was 7.1% in 2018, 7.9% in 2019, 11.3% in 2020, and 11.9% in 2021. The percentage of T4b patients who received LDG in August was 0.9% in 2018, 0.6% in 2019, 0.9% in 2020, and 1.1% in 2021. The percentage of N3b patients who received LDG in August was 0.8% in 2018, 1.6% in 2019, 0.9% in 2020, and 2.2% in 2021. Among patients who received LLAR, the percentage who received LLAR with preoperative chemotherapy in August was 7.9% in 2018, 7.3% in 2019, 11.0% in 2020, and 10.7% in 2021. The percentage of T4b patients who received LLAR in August was 3.2% in 2018, 2.5% in 2019, 3.7% in 2019, and 3.4% in 2021. Table S1A,B show the patient backgrounds for LDG and LLAR by month from 2018 to 2021.

### 3.3 Trends in the annual ratios of standardized mortality (2018–2021)

**FIGURE 3:**
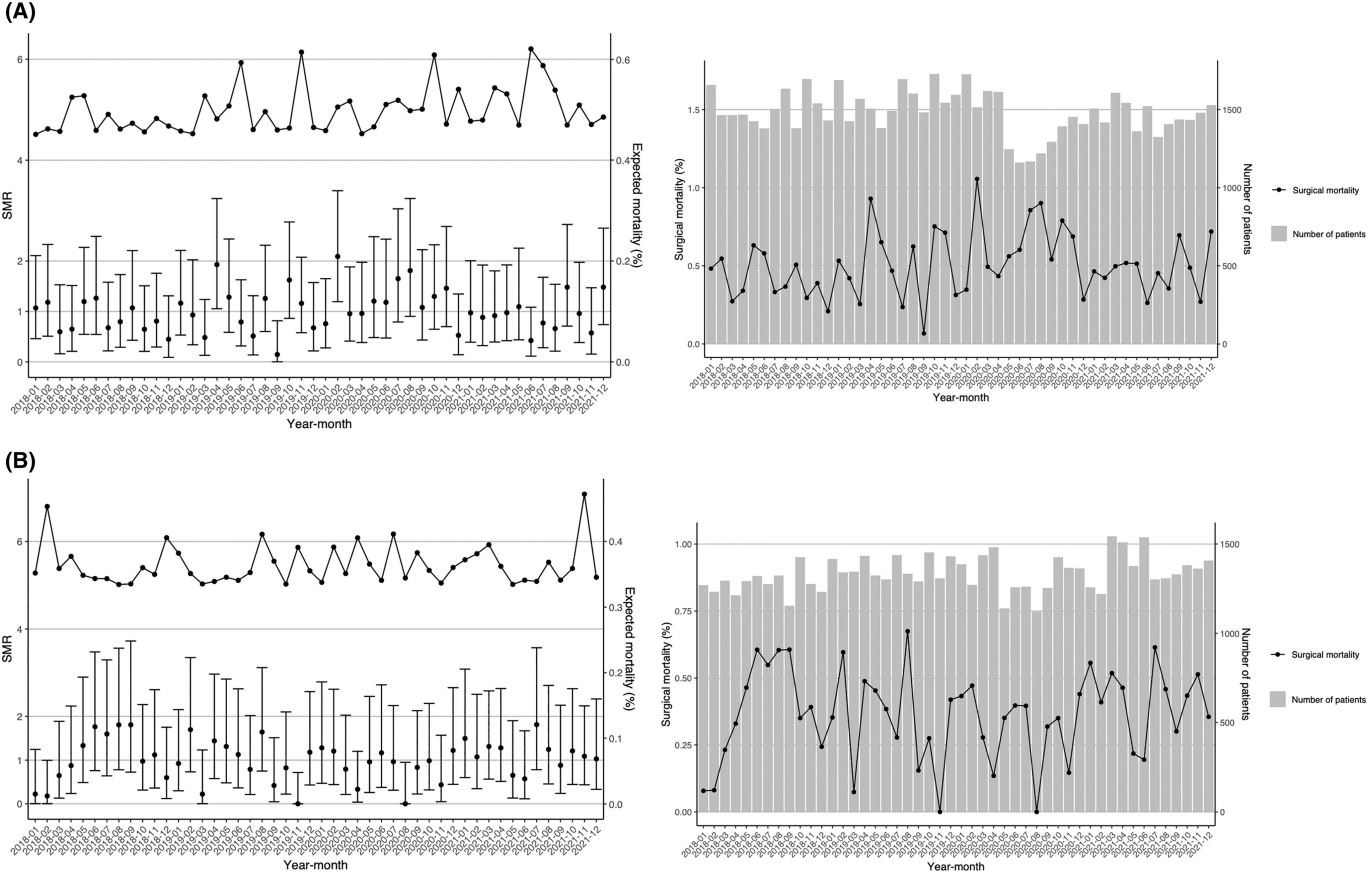
Mortality (standardized mortality ratio). The standardized mortality ratio (SMR) during each month for (A) LDG and (B) LLAR. Vertical lines indicate the SMR with the 95% confidence interval. Dashed lines indicate the expected mortality rate.

Figure 3A,B show the standardized mortality ratio of LDG and LLAR, respectively. The expected morality of LDG was approximately 0.5, which was as low as in the previous year and in the pre‐pandemic period. The LLAR also remained at approximately 0.35, which was as low as in the previous year and before the pandemic.

### 3.4 Trends in the monthly ratios of standardized anastomotic leakage (2018–2021)

**FIGURE 4:**
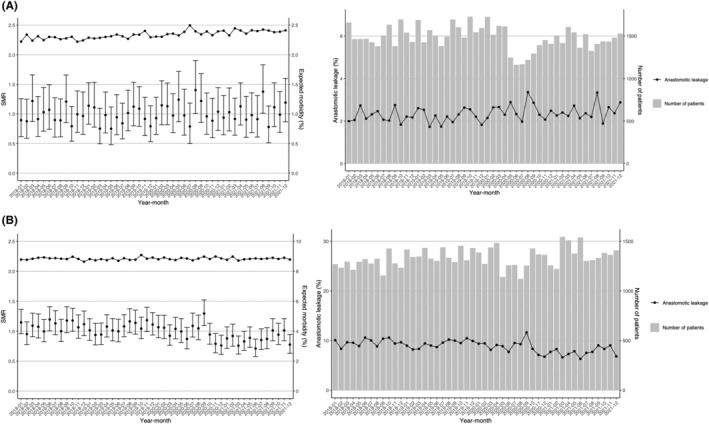
Morbidity (anastomotic leakage) (standardized morbidity ratio). The standardized morbidity ratio (SMR) for anastomotic leakage during each month for (A) LDG and (B) LLAR. Vertical lines indicate the SMR with the 95% confidence interval. Dashed lines indicate the expected morbidity rate.

Next, we examined the incidence of postoperative anastomotic leakage, which is an important complication. The mean actual incidence rates of anastomotic leakage of LDG and LLAR during the study period were 2.3% and 8.8%, respectively. We then calculated the expected incidence rates adjusted for various patient risks and obtained the SMRs accordingly. Figure 4A,B show the trends of the expected morbidity rate and SMR for anastomotic leakage in patients who underwent LDG and LLAR, respectively. During the study period, the incidence of anastomotic leakage did not change and remained at the same level as before the pandemic in LDG, while a downward trend was observed in 2021 for LLAR in comparison to before the pandemic.

## 4 DISCUSSION

In this study, we reported the impact of COVID‐19 on laparoscopic surgery in Japan using the NCD database from the early stages of the pandemic in 2020–2021, when the infection was widespread. The present study analyzed postoperative mortality and anastomotic leakage, two of the major complications, with risk adjustment. The study included changes in the number of laparoscopic surgeries (LDG and LLAR). According to the results of this study using the NCD, LDG for gastric cancer patients and LLAR for rectal cancer could be safely implemented during a pandemic without increasing mortality or morbidity, two of the most important complications. In both procedures, the rates of these complications were as low as they were before the pandemic. Although laparoscopic surgery is associated with its own concerns, such as preparation of surgical instruments and infection by aerosols, the quality of laparoscopic surgery in LDG and LLAR was considered to be as high as it was before the pandemic. To our knowledge, this is the first report to clarify the risk‐adjusted outcomes of laparoscopic surgery for gastric and rectal cancer performed during the COVID‐19 pandemic.

There are several reasons as to why the short‐term outcomes of surgery, mortality, and anastomotic leakage rates, did not worsen during the pandemic. First, the high operative quality of laparoscopic surgery for gastric and rectal cancer in Japan was maintained due to the appropriate spread and successful establishment of the technique in Japan. Therefore, despite limited medical resources, the short‐term results may have remained good, without worsening. In Japan, based on the announcements and guidelines of the relevant surgical societies, infection prevention measures and restrictions and the appropriate use of surgical‐related equipment and infection control equipment were indicated, and many hospitals followed their policies to prevent outcomes from worsening.^5^, ^16^, ^17^ Second, the fact that laparoscopic surgery for gastric cancer and rectal cancer is less invasive than laparoscopic surgery for liver and pancreatic cancer may explain why the short‐term surgical outcomes were not significantly affected by the spread of infection. Similar studies of more invasive laparoscopic procedures, such as laparoscopic surgery for liver cancer and pancreatic cancer, are needed in the future. Third, screening for COVID‐19 infection in patients before gastric and rectal cancer surgery to avoid surgery in COVID‐19‐positive cases may have been appropriately performed at each institution in Japan.

The total number of gastric and rectal cancer surgeries declined. The details of this trend were as follows: open surgery for both types of procedure declined, whereas laparoscopic surgery declined in 2020 and remained low in 2021 without recovery. The trend in the number of surgeries for each type of procedure did not differ to a statistically significant extent between the high infection and low infection regions. Robotic surgery, on the other hand, showed an increasing trend. One possible explanation for this trend may be that robotic surgery is now covered by national medical insurance and its application is currently expanding in Japan. The trend of choosing laparoscopic surgery and robotic surgery, which are less invasive surgical approaches, over open surgery has been observed, and we believe that the decrease in the number of surgeries and restrictions due to the spread of COVID‐19 have not had any impact on the trend in the selection of surgical approaches.

The decrease in visits for early‐stage cancer detection triggered by health checkups and physical examinations is thought to be one of the factors that led to the decrease in surgery for early‐stage cancers. In fact, there have been reports from Japan and abroad indicating that the incidence of advanced cancer is on the rise due to the decline in the number of endoscopic gastrointestinal examinations.^18^, ^19^, ^20^, ^21^ It is undeniable that the detection of gastric and rectal cancer may be delayed until the appearance of obvious or serious symptoms due to the skipping of medical examinations or tests that might have been recommended if the patient had visited a medical institution. This trend is expected to continue after 2021, and future trends in the patient population need to be monitored closely. The increase in the number of advanced cancers and the limited number of surgeries performed have led to an increase in preoperative treatment in 2020 and 2021.

The present study is associated with several limitations. First, the number of patients with COVID‐19 who were included in the study population was unknown, and we cannot evaluate the effect of COVID‐19 on postoperative complications. However, there seem to be fewer cases of elective surgery in patients with COVID‐19 because preoperative screening is performed frequently in Japan. Second, all cases of emergency surgery were excluded, and the present data can only be applied to elective surgeries. Emergency surgery may be needed for locally advanced cancer associated with bleeding or stenosis. Therefore, we could not evaluate the surgical outcomes for all cases of locally advanced cancer. Third, the patients were divided into two groups according to the degree of infection; however, it will be necessary to evaluate three groups by adding a moderately infected area. Fourth, the present study could not be performed using pathology outcome data. Future studies will be needed to examine this issue. Fifth, it is unknown whether the healthcare providers were infected, and hence the effect of aerosols in laparoscopic surgery is unknown. Finally, long‐term outcomes were not investigated in this study. It is considered that prognostic effect of the delay of surgical treatment due to triage will appear after several years. Thus, further studies are needed to precisely evaluate the impact of the COVID‐19 pandemic on the outcomes of surgery for gastric and rectal cancer.

In conclusion, laparoscopic surgery was performed safely for gastric and rectal cancer in Japan and was not affected by the COVID‐19 pandemic. More evidence, such as long‐term outcomes, is needed to understand who laparoscopic surgery can be performed on safely during future pandemics. In 2022, the COVID‐19 pandemic is expected to continue to grow, and the impact on surgical care may be even greater and should be closely monitored and evaluated to determine its impact.

## AUTHOR CONTRIBUTIONS

Study concepts, design, and data interpretation: all authors. Data acquisition and analysis: Hideki Endo, Hiroyuki Yamamoto. Article preparation: all authors. Article review: Yoshihiro Kakeji, Yuko Kitagawa, Akinobu Taketomi, Masaki Mori.

## CONFLICT OF INTEREST STATEMENT

Hiroyuki Yamamoto and Hideki Endo are affiliated with the Department of Healthcare Quality Assessment at the University of Tokyo. The department is a social collaboration department supported by grants from the National Clinical Database, Intuitive Surgical Sarl, Johnson & Johnson K.K., and Nipro Co. Masafumi Inomata is an Editorial Board Member of *Annals of Gastroenterological Surgery* dealing with the lower digestive tract. Susumu Eguchi is an Editorial Board Member of *Annals of Gastroenterological Surgery* dealing with the hepato‐biliary‐pancreatic. Yukinori Kurokawa is an Associate Editor of the *Annals of Gastroenterological Surgery* dealing with the upper digestive tract, and has received lecture fees from Johnson & Johnson, Covidien Japan, Stryker, and MC Medical outside of the submitted work. Yoshihiko Kakeji is an Associate Editor of *Annals of Gastroenterological Surgery* dealing with the lower digestive tract. Yuko Kitagawa is an Editor‐in‐Chief of *Annals of Gastroenterological Surgery*. Masaki Mori is an Emeritus Editor‐in‐Chief of *Annals of Gastroenterological Surgery*. The remaining authors declare no conflicts of interest for this article.

## ETHICS STATEMENTS

Approval of research protocol: The study protocol was approved by the institutional review board of Oita University (approval number: 2444). Informed consent: N/A. Registry and the Registration No. of the study/trial: N/A. Animal Studies: N/A.

## Supporting information


Table S1.
