# Supplementary material for: Investigation of the impact of COVID‐19 on postoperative outcomes using a nationwide Japanese database of patients undergoing laparoscopic distal gastrectomy and low anterior resection for gastric cancer and rectal cancer
Source: Ann Gastroenterol Surg. 2024 Jan 28;8(3):521–9. doi: 10.1002/ags3.12776 (PMC11066483; doi:10.1002/ags3.12776)
Supplement: Supplementary file 1 — Table S1. [file AGS3-8-521-s001.docx]

Supplement 1a Background of patients with LDG

| 2018 | 1 | 2 | 3 | 4 | 5 | 6 | 7 | 8 | 9 | 10 | 11 | 12 |
| --- | --- | --- | --- | --- | --- | --- | --- | --- | --- | --- | --- | --- |
| n | 1659 | 1465 | 1465 | 1468 | 1426 | 1381 | 1505 | 1634 | 1381 | 1698 | 1540 | 1432 |
| age (median [IQR]) | 70  [64, 77] | 70  [64, 77] | 71  [64, 77] | 71  [64, 79] | 71  [64, 77] | 71  [65, 78] | 71  [64, 78] | 71  [64, 78] | 71  [64, 77] | 71  [64, 78] | 71  [64, 78] | 71  [64, 77] |
| category of age (%) |  |  |  |  |  |  |  |  |  |  |  |  |
| <65 | 447 (26.9) | 392 (26.8) | 370 (25.3) | 379 (25.8) | 362 (25.4) | 324 (23.5) | 377 (25.0) | 414 (25.3) | 369 (26.7) | 440 (25.9) | 423 (27.5) | 385 (26.9) |
| 65-75 | 706 (42.6) | 612 (41.8) | 636 (43.4) | 555 (37.8) | 602 (42.2) | 571 (41.3) | 626 (41.6) | 661 (40.5) | 550 (39.8) | 684 (40.3) | 581 (37.7) | 578 (40.4) |
| 75< | 506 (30.5) | 461 (31.5) | 459 (31.3) | 534 (36.4) | 462 (32.4) | 486 (35.2) | 502 (33.4) | 559 (34.2) | 462 (33.5) | 574 (33.8) | 536 (34.8) | 469 (32.8) |
| male (%) | 1040 (62.7) | 962 (65.7) | 946 (64.6) | 943 (64.2) | 948 (66.5) | 929 (67.3) | 965 (64.1) | 1095 (67.0) | 923 (66.8) | 1134 (66.8) | 998 (64.8) | 920 (64.2) |
| BMI≥25 (%) | 377 (22.7) | 367 (25.1) | 337 (23.0) | 340 (23.2) | 327 (22.9) | 291 (21.1) | 318 (21.1) | 363 (22.2) | 310 (22.4) | 380 (22.4) | 344 (22.3) | 314 (21.9) |
| Preoperative chemotherapy (%) | 17 (1.0) | 22 (1.5) | 27 (1.8) | 22 (1.5) | 22 (1.5) | 26 (1.9) | 28 (1.9) | 26 (1.6) | 20 (1.4) | 26 (1.5) | 24 (1.6) | 15 (1.0) |
| DM (%) | 262 (15.8) | 219 (14.9) | 236 (16.1) | 243 (16.6) | 223 (15.6) | 213 (15.4) | 271 (18.0) | 285 (17.4) | 250 (18.1) | 282 (16.6) | 240 (15.6) | 242 (16.9) |
| Brinkman index (%) |  |  |  |  |  |  |  |  |  |  |  |  |
| 0 | 855 (51.5) | 731 (49.9) | 742 (50.6) | 747 (50.9) | 710 (49.8) | 660 (47.8) | 730 (48.5) | 784 (48.0) | 676 (49.0) | 806 (47.5) | 777 (50.5) | 726 (50.7) |
| <400 | 240 (14.5) | 215 (14.7) | 196 (13.4) | 196 (13.4) | 208 (14.6) | 197 (14.3) | 222 (14.8) | 260 (15.9) | 187 (13.5) | 260 (15.3) | 201 (13.1) | 223 (15.6) |
| ≥400 | 564 (34.0) | 519 (35.4) | 527 (36.0) | 525 (35.8) | 508 (35.6) | 524 (37.9) | 553 (36.7) | 590 (36.1) | 518 (37.5) | 632 (37.2) | 562 (36.5) | 483 (33.7) |
| Habitual alcohol intake (%) | 539 (32.5) | 463 (31.6) | 491 (33.5) | 480 (32.7) | 481 (33.7) | 454 (32.9) | 502 (33.4) | 538 (32.9) | 482 (34.9) | 582 (34.3) | 527 (34.2) | 485 (33.9) |
| COPD (%) | 76 (4.6) | 70 (4.8) | 73 (5.0) | 62 (4.2) | 73 (5.1) | 72 (5.2) | 70 (4.7) | 84 (5.1) | 61 (4.4) | 91 (5.4) | 66 (4.3) | 66 (4.6) |
| Congestive heart disease (%) | 8 (0.5) | 8 (0.5) | 6 (0.4) | 2 (0.1) | 7 (0.5) | 9 (0.7) | 4 (0.3) | 4 (0.2) | 7 (0.5) | 2 (0.1) | 0 (0.0) | 5 (0.3) |
| Ischemic heart disease (%) | 21 (1.3) | 18 (1.2) | 8 (0.5) | 18 (1.2) | 16 (1.1) | 21 (1.5) | 22 (1.5) | 15 (0.9) | 17 (1.2) | 23 (1.4) | 10 (0.6) | 14 (1.0) |
| Hypertension(%) | 681 (41.0) | 591 (40.3) | 566 (38.6) | 630 (42.9) | 570 (40.0) | 623 (45.1) | 647 (43.0) | 702 (43.0) | 589 (42.7) | 743 (43.8) | 639 (41.5) | 592 (41.3) |
| Dialysis (%) | 7 (0.4) | 8 (0.5) | 12 (0.8) | 7 (0.5) | 7 (0.5) | 9 (0.7) | 16 (1.1) | 8 (0.5) | 10 (0.7) | 9 (0.5) | 8 (0.5) | 7 (0.5) |
| Cerebrovascular disease (%) | 51 (3.1) | 42 (2.9) | 39 (2.7) | 60 (4.1) | 56 (3.9) | 48 (3.5) | 67 (4.5) | 70 (4.3) | 36 (2.6) | 54 (3.2) | 54 (3.5) | 49 (3.4) |
| Steroid (%) | 14 (0.8) | 10 (0.7) | 15 (1.0) | 19 (1.3) | 15 (1.1) | 11 (0.8) | 18 (1.2) | 23 (1.4) | 14 (1.0) | 15 (0.9) | 20 (1.3) | 14 (1.0) |
| Weight loss (%) | 15 (0.9) | 22 (1.5) | 20 (1.4) | 33 (2.2) | 20 (1.4) | 36 (2.6) | 26 (1.7) | 30 (1.8) | 19 (1.4) | 31 (1.8) | 24 (1.6) | 33 (2.3) |
| Bleeding disorder (%) | 160 (9.6) | 148 (10.1) | 131 (8.9) | 165 (11.2) | 164 (11.5) | 171 (12.4) | 180 (12.0) | 205 (12.5) | 155 (11.2) | 191 (11.2) | 162 (10.5) | 141 (9.8) |
| Preoperative transfusion (%) | 12 (0.7) | 13 (0.9) | 14 (1.0) | 15 (1.0) | 8 (0.6) | 23 (1.7) | 15 (1.0) | 12 (0.7) | 17 (1.2) | 17 (1.0) | 13 (0.8) | 20 (1.4) |
| ASA PS (%) |  |  |  |  |  |  |  |  |  |  |  |  |
| ASA1 | 366 (22.1) | 326 (22.3) | 308 (21.0) | 280 (19.1) | 255 (17.9) | 254 (18.4) | 256 (17.0) | 320 (19.6) | 253 (18.3) | 301 (17.7) | 339 (22.0) | 301 (21.0) |
| ASA2 | 1134 (68.4) | 976 (66.6) | 1013 (69.1) | 1023 (69.7) | 1012 (71.0) | 966 (69.9) | 1063 (70.6) | 1128 (69.0) | 978 (70.8) | 1226 (72.2) | 1044 (67.8) | 1005 (70.2) |
| ASA3_5 | 159 (9.6) | 163 (11.1) | 144 (9.8) | 165 (11.2) | 159 (11.2) | 161 (11.7) | 186 (12.4) | 186 (11.4) | 150 (10.9) | 171 (10.1) | 157 (10.2) | 126 (8.8) |
| T factor (%) |  |  |  |  |  |  |  |  |  |  |  |  |
| T0 | 5 (0.3) | 2 (0.1) | 4 (0.3) | 3 (0.2) | 6 (0.4) | 2 (0.1) | 5 (0.3) | 6 (0.4) | 6 (0.4) | 6 (0.4) | 7 (0.5) | 1 (0.1) |
| Tis | 16 (1.0) | 13 (0.9) | 9 (0.6) | 11 (0.7) | 6 (0.4) | 7 (0.5) | 6 (0.4) | 5 (0.3) | 7 (0.5) | 6 (0.4) | 9 (0.6) | 7 (0.5) |
| T1a | 425 (25.6) | 381 (26.0) | 372 (25.4) | 363 (24.7) | 333 (23.4) | 302 (21.9) | 364 (24.2) | 391 (23.9) | 351 (25.4) | 373 (22.0) | 333 (21.6) | 329 (23.0) |
| T1b | 751 (45.3) | 651 (44.4) | 645 (44.0) | 606 (41.3) | 642 (45.0) | 580 (42.0) | 653 (43.4) | 702 (43.0) | 568 (41.1) | 776 (45.7) | 700 (45.5) | 598 (41.8) |
| T2 | 216 (13.0) | 170 (11.6) | 184 (12.6) | 185 (12.6) | 169 (11.9) | 221 (16.0) | 190 (12.6) | 210 (12.9) | 202 (14.6) | 227 (13.4) | 201 (13.1) | 198 (13.8) |
| T3 | 152 (9.2) | 134 (9.1) | 138 (9.4) | 178 (12.1) | 157 (11.0) | 158 (11.4) | 165 (11.0) | 192 (11.8) | 137 (9.9) | 179 (10.5) | 156 (10.1) | 185 (12.9) |
| T4a | 83 (5.0) | 105 (7.2) | 102 (7.0) | 107 (7.3) | 108 (7.6) | 101 (7.3) | 116 (7.7) | 119 (7.3) | 104 (7.5) | 120 (7.1) | 120 (7.8) | 101 (7.1) |
| T4b | 11 (0.7) | 9 (0.6) | 11 (0.8) | 15 (1.0) | 5 (0.4) | 10 (0.7) | 6 (0.4) | 9 (0.6) | 6 (0.4) | 11 (0.6) | 14 (0.9) | 13 (0.9) |
| N factor (%) |  |  |  |  |  |  |  |  |  |  |  |  |
| N0 | 1357 (81.8) | 1151 (78.6) | 1167 (79.7) | 1148 (78.2) | 1111 (77.9) | 1042 (75.5) | 1183 (78.6) | 1284 (78.6) | 1080 (78.2) | 1337 (78.7) | 1221 (79.3) | 1124 (78.5) |
| N1 | 167 (10.1) | 159 (10.9) | 150 (10.2) | 168 (11.4) | 146 (10.2) | 182 (13.2) | 161 (10.7) | 174 (10.6) | 155 (11.2) | 183 (10.8) | 156 (10.1) | 141 (9.8) |
| N2 | 87 (5.2) | 95 (6.5) | 99 (6.8) | 91 (6.2) | 110 (7.7) | 93 (6.7) | 91 (6.0) | 110 (6.7) | 86 (6.2) | 115 (6.8) | 94 (6.1) | 97 (6.8) |
| N3a | 42 (2.5) | 47 (3.2) | 38 (2.6) | 40 (2.7) | 43 (3.0) | 49 (3.5) | 54 (3.6) | 56 (3.4) | 46 (3.3) | 52 (3.1) | 50 (3.2) | 48 (3.4) |
| N3b | 6 (0.4) | 13 (0.9) | 11 (0.8) | 21 (1.4) | 16 (1.1) | 15 (1.1) | 16 (1.1) | 10 (0.6) | 14 (1.0) | 11 (0.6) | 19 (1.2) | 22 (1.5) |
| M1 (%) | 19 (1.1) | 23 (1.6) | 18 (1.2) | 24 (1.6) | 17 (1.2) | 25 (1.8) | 23 (1.5) | 20 (1.2) | 22 (1.6) | 21 (1.2) | 23 (1.5) | 21 (1.5) |
|  |  |  |  |  |  |  |  |  |  |  |  |  |
| 2019 | 1 | 2 | 3 | 4 | 5 | 6 | 7 | 8 | 9 | 10 | 11 | 12 |
| n | 1689 | 1426 | 1569 | 1506 | 1382 | 1493 | 1695 | 1603 | 1482 | 1728 | 1544 | 1595 |
| age (median [IQR]) | 71  [64, 77] | 71  [65, 78] | 71  [64, 78] | 71  [64, 78] | 72  [65, 78] | 72  [66, 79] | 71  [64, 78] | 72  [65, 79] | 72  [65, 79] | 72  [65, 78] | 72  [66, 79] | 71  [65, 78] |
| category of age (%) |  |  |  |  |  |  |  |  |  |  |  |  |
| <65 | 437 (25.9) | 339 (23.8) | 399 (25.4) | 382 (25.4) | 334 (24.2) | 337 (22.6) | 458 (27.0) | 379 (23.6) | 332 (22.4) | 398 (23.0) | 324 (21.0) | 391 (24.5) |
| 65-75 | 713 (42.2) | 606 (42.5) | 636 (40.5) | 612 (40.6) | 552 (39.9) | 583 (39.0) | 679 (40.1) | 629 (39.2) | 586 (39.5) | 729 (42.2) | 645 (41.8) | 641 (40.2) |
| 75< | 539 (31.9) | 481 (33.7) | 534 (34.0) | 512 (34.0) | 496 (35.9) | 573 (38.4) | 558 (32.9) | 595 (37.1) | 564 (38.1) | 601 (34.8) | 575 (37.2) | 563 (35.3) |
| male (%) | 1112 (65.8) | 918 (64.4) | 1006 (64.1) | 964 (64.0) | 894 (64.7) | 964 (64.6) | 1140 (67.3) | 1076 (67.1) | 1008 (68.0) | 1215 (70.3) | 1029 (66.6) | 1012 (63.4) |
| BMI≥25 (%) | 422 (25.0) | 340 (23.8) | 371 (23.6) | 370 (24.6) | 316 (22.9) | 327 (21.9) | 391 (23.1) | 333 (20.8) | 333 (22.5) | 397 (23.0) | 346 (22.4) | 355 (22.3) |
| Preoperative chemotherapy (%) | 31 (1.8) | 17 (1.2) | 28 (1.8) | 31 (2.1) | 28 (2.0) | 20 (1.3) | 20 (1.2) | 23 (1.4) | 32 (2.2) | 34 (2.0) | 19 (1.2) | 32 (2.0) |
| DM (%) | 300 (17.8) | 252 (17.7) | 269 (17.1) | 265 (17.6) | 225 (16.3) | 263 (17.6) | 262 (15.5) | 283 (17.7) | 250 (16.9) | 303 (17.5) | 287 (18.6) | 239 (15.0) |
| Brinkman index (%) |  |  |  |  |  |  |  |  |  |  |  |  |
| 0 | 810 (48.0) | 691 (48.5) | 791 (50.4) | 736 (48.9) | 701 (50.7) | 735 (49.2) | 809 (47.7) | 777 (48.5) | 708 (47.8) | 794 (45.9) | 723 (46.8) | 760 (47.6) |
| <400 | 271 (16.0) | 215 (15.1) | 243 (15.5) | 227 (15.1) | 201 (14.5) | 201 (13.5) | 264 (15.6) | 232 (14.5) | 229 (15.5) | 266 (15.4) | 225 (14.6) | 242 (15.2) |
| ≥400 | 608 (36.0) | 520 (36.5) | 535 (34.1) | 543 (36.1) | 480 (34.7) | 557 (37.3) | 622 (36.7) | 594 (37.1) | 545 (36.8) | 668 (38.7) | 596 (38.6) | 593 (37.2) |
| Habitual alcohol intake (%) | 570 (33.7) | 477 (33.5) | 489 (31.2) | 503 (33.4) | 434 (31.4) | 499 (33.4) | 559 (33.0) | 532 (33.2) | 482 (32.5) | 596 (34.5) | 528 (34.2) | 506 (31.7) |
| COPD (%) | 73 (4.3) | 76 (5.3) | 80 (5.1) | 87 (5.8) | 78 (5.6) | 72 (4.8) | 77 (4.5) | 86 (5.4) | 74 (5.0) | 81 (4.7) | 76 (4.9) | 84 (5.3) |
| Congestive heart disease (%) | 1 (0.1) | 7 (0.5) | 8 (0.5) | 11 (0.7) | 6 (0.4) | 13 (0.9) | 14 (0.8) | 5 (0.3) | 4 (0.3) | 6 (0.3) | 11 (0.7) | 3 (0.2) |
| Ischemic heart disease (%) | 15 (0.9) | 24 (1.7) | 18 (1.1) | 26 (1.7) | 20 (1.4) | 25 (1.7) | 23 (1.4) | 21 (1.3) | 18 (1.2) | 17 (1.0) | 14 (0.9) | 17 (1.1) |
| Hypertension(%) | 754 (44.6) | 640 (44.9) | 637 (40.6) | 646 (42.9) | 642 (46.5) | 690 (46.2) | 743 (43.8) | 732 (45.7) | 669 (45.1) | 779 (45.1) | 717 (46.4) | 693 (43.4) |
| Dialysis (%) | 18 (1.1) | 8 (0.6) | 9 (0.6) | 7 (0.5) | 13 (0.9) | 16 (1.1) | 14 (0.8) | 4 (0.2) | 6 (0.4) | 12 (0.7) | 9 (0.6) | 7 (0.4) |
| Cerebrovascular disease (%) | 63 (3.7) | 54 (3.8) | 50 (3.2) | 46 (3.1) | 55 (4.0) | 79 (5.3) | 56 (3.3) | 70 (4.4) | 67 (4.5) | 64 (3.7) | 62 (4.0) | 62 (3.9) |
| Steroid (%) | 21 (1.2) | 11 (0.8) | 15 (1.0) | 13 (0.9) | 15 (1.1) | 20 (1.3) | 21 (1.2) | 17 (1.1) | 11 (0.7) | 15 (0.9) | 20 (1.3) | 20 (1.3) |
| Weight loss (%) | 31 (1.8) | 29 (2.0) | 36 (2.3) | 25 (1.7) | 31 (2.2) | 25 (1.7) | 38 (2.2) | 29 (1.8) | 36 (2.4) | 25 (1.4) | 38 (2.5) | 36 (2.3) |
| Bleeding disorder (%) | 196 (11.6) | 170 (11.9) | 206 (13.1) | 181 (12.0) | 190 (13.7) | 211 (14.1) | 190 (11.2) | 200 (12.5) | 202 (13.6) | 191 (11.1) | 203 (13.1) | 197 (12.4) |
| Preoperative transfusion (%) | 16 (0.9) | 13 (0.9) | 15 (1.0) | 17 (1.1) | 14 (1.0) | 20 (1.3) | 15 (0.9) | 9 (0.6) | 14 (0.9) | 13 (0.8) | 20 (1.3) | 21 (1.3) |
| ASA PS (%) |  |  |  |  |  |  |  |  |  |  |  |  |
| ASA1 | 321 (19.0) | 254 (17.8) | 299 (19.1) | 293 (19.5) | 248 (17.9) | 211 (14.1) | 296 (17.5) | 277 (17.3) | 248 (16.7) | 314 (18.2) | 260 (16.8) | 286 (17.9) |
| ASA2 | 1175 (69.6) | 1014 (71.1) | 1093 (69.7) | 1032 (68.5) | 969 (70.1) | 1064 (71.3) | 1204 (71.0) | 1138 (71.0) | 1050 (70.9) | 1225 (70.9) | 1108 (71.8) | 1127 (70.7) |
| ASA3_5 | 193 (11.4) | 158 (11.1) | 177 (11.3) | 181 (12.0) | 165 (11.9) | 218 (14.6) | 195 (11.5) | 188 (11.7) | 184 (12.4) | 189 (10.9) | 176 (11.4) | 182 (11.4) |
| T factor (%) |  |  |  |  |  |  |  |  |  |  |  |  |
| T0 | 7 (0.4) | 4 (0.3) | 7 (0.4) | 11 (0.7) | 3 (0.2) | 3 (0.2) | 5 (0.3) | 6 (0.4) | 4 (0.3) | 4 (0.2) | 2 (0.1) | 5 (0.3) |
| Tis | 13 (0.8) | 6 (0.4) | 10 (0.6) | 7 (0.5) | 7 (0.5) | 3 (0.2) | 12 (0.7) | 7 (0.4) | 8 (0.5) | 3 (0.2) | 11 (0.7) | 4 (0.3) |
| T1a | 385 (22.8) | 316 (22.2) | 346 (22.1) | 343 (22.8) | 303 (21.9) | 337 (22.6) | 376 (22.2) | 348 (21.7) | 325 (21.9) | 353 (20.4) | 315 (20.4) | 331 (20.8) |
| T1b | 756 (44.8) | 632 (44.3) | 672 (42.8) | 646 (42.9) | 615 (44.5) | 624 (41.8) | 719 (42.4) | 678 (42.3) | 650 (43.9) | 791 (45.8) | 653 (42.3) | 679 (42.6) |
| T2 | 244 (14.4) | 197 (13.8) | 204 (13.0) | 224 (14.9) | 171 (12.4) | 191 (12.8) | 222 (13.1) | 218 (13.6) | 196 (13.2) | 227 (13.1) | 234 (15.2) | 208 (13.0) |
| T3 | 166 (9.8) | 157 (11.0) | 197 (12.6) | 153 (10.2) | 165 (11.9) | 177 (11.9) | 214 (12.6) | 212 (13.2) | 154 (10.4) | 196 (11.3) | 201 (13.0) | 208 (13.0) |
| T4a | 108 (6.4) | 101 (7.1) | 113 (7.2) | 113 (7.5) | 111 (8.0) | 143 (9.6) | 137 (8.1) | 124 (7.7) | 134 (9.0) | 137 (7.9) | 119 (7.7) | 148 (9.3) |
| T4b | 10 (0.6) | 13 (0.9) | 20 (1.3) | 9 (0.6) | 7 (0.5) | 15 (1.0) | 10 (0.6) | 10 (0.6) | 11 (0.7) | 17 (1.0) | 9 (0.6) | 12 (0.8) |
| N factor (%) |  |  |  |  |  |  |  |  |  |  |  |  |
| N0 | 1368 (81.0) | 1132 (79.4) | 1212 (77.2) | 1186 (78.8) | 1072 (77.6) | 1136 (76.1) | 1286 (75.9) | 1231 (76.8) | 1159 (78.2) | 1346 (77.9) | 1174 (76.0) | 1216 (76.2) |
| N1 | 161 (9.5) | 150 (10.5) | 185 (11.8) | 168 (11.2) | 153 (11.1) | 162 (10.9) | 199 (11.7) | 177 (11.0) | 148 (10.0) | 187 (10.8) | 174 (11.3) | 175 (11.0) |
| N2 | 108 (6.4) | 84 (5.9) | 93 (5.9) | 76 (5.0) | 90 (6.5) | 115 (7.7) | 120 (7.1) | 120 (7.5) | 105 (7.1) | 123 (7.1) | 118 (7.6) | 118 (7.4) |
| N3a | 41 (2.4) | 43 (3.0) | 54 (3.4) | 53 (3.5) | 48 (3.5) | 58 (3.9) | 67 (4.0) | 57 (3.6) | 56 (3.8) | 52 (3.0) | 55 (3.6) | 66 (4.1) |
| N3b | 11 (0.7) | 17 (1.2) | 25 (1.6) | 23 (1.5) | 19 (1.4) | 22 (1.5) | 23 (1.4) | 18 (1.1) | 14 (0.9) | 20 (1.2) | 23 (1.5) | 20 (1.3) |
| M1 (%) | 24 (1.4) | 28 (2.0) | 29 (1.8) | 23 (1.5) | 28 (2.0) | 31 (2.1) | 27 (1.6) | 27 (1.7) | 19 (1.3) | 24 (1.4) | 18 (1.2) | 26 (1.6) |
|  |  |  |  |  |  |  |  |  |  |  |  |  |
| 2020 | 1 | 2 | 3 | 4 | 5 | 6 | 7 | 8 | 9 | 10 | 11 | 12 |
| n | 1726 | 1517 | 1621 | 1614 | 1247 | 1162 | 1168 | 1221 | 1294 | 1393 | 1453 | 1408 |
| age (median [IQR]) | 71  [64, 78] | 72  [65, 78] | 72  [66, 79] | 71  [65, 78] | 72  [66, 79] | 72  [65, 79] | 73  [67, 80] | 72  [66, 79] | 73  [65, 78] | 73  [66, 79] | 72  [66, 79] | 72  [66, 78] |
| category of age (%) |  |  |  |  |  |  |  |  |  |  |  |  |
| <65 | 454 (26.3) | 359 (23.7) | 347 (21.4) | 402 (24.9) | 259 (20.8) | 253 (21.8) | 235 (20.1) | 272 (22.3) | 298 (23.0) | 319 (22.9) | 319 (22.0) | 311 (22.1) |
| 65-75 | 719 (41.7) | 603 (39.7) | 672 (41.5) | 656 (40.6) | 517 (41.5) | 460 (39.6) | 459 (39.3) | 475 (38.9) | 497 (38.4) | 527 (37.8) | 584 (40.2) | 581 (41.3) |
| 75< | 553 (32.0) | 555 (36.6) | 602 (37.1) | 556 (34.4) | 471 (37.8) | 449 (38.6) | 474 (40.6) | 474 (38.8) | 499 (38.6) | 547 (39.3) | 550 (37.9) | 516 (36.6) |
| male (%) | 1111 (64.4) | 1008 (66.4) | 1088 (67.1) | 1088 (67.4) | 801 (64.2) | 793 (68.2) | 794 (68.0) | 809 (66.3) | 867 (67.0) | 955 (68.6) | 942 (64.8) | 919 (65.3) |
| BMI≥25 (%) | 426 (24.7) | 347 (22.9) | 426 (26.3) | 388 (24.0) | 307 (24.6) | 253 (21.8) | 285 (24.4) | 268 (21.9) | 294 (22.7) | 316 (22.7) | 324 (22.3) | 342 (24.3) |
| Preoperative chemotherapy (%) | 37 (2.1) | 23 (1.5) | 32 (2.0) | 38 (2.4) | 34 (2.7) | 40 (3.4) | 18 (1.5) | 33 (2.7) | 34 (2.6) | 36 (2.6) | 29 (2.0) | 28 (2.0) |
| DM (%) | 322 (18.7) | 276 (18.2) | 280 (17.3) | 296 (18.3) | 230 (18.4) | 236 (20.3) | 211 (18.1) | 239 (19.6) | 262 (20.2) | 257 (18.4) | 284 (19.5) | 251 (17.8) |
| Brinkman index (%) |  |  |  |  |  |  |  |  |  |  |  |  |
| 0 | 854 (49.5) | 733 (48.3) | 770 (47.5) | 726 (45.0) | 618 (49.6) | 524 (45.1) | 515 (44.1) | 531 (43.5) | 612 (47.3) | 611 (43.9) | 692 (47.6) | 676 (48.0) |
| <400 | 234 (13.6) | 223 (14.7) | 249 (15.4) | 256 (15.9) | 187 (15.0) | 171 (14.7) | 179 (15.3) | 217 (17.8) | 194 (15.0) | 229 (16.4) | 221 (15.2) | 213 (15.1) |
| ≥400 | 638 (37.0) | 561 (37.0) | 602 (37.1) | 632 (39.2) | 442 (35.4) | 467 (40.2) | 474 (40.6) | 473 (38.7) | 488 (37.7) | 553 (39.7) | 540 (37.2) | 519 (36.9) |
| Habitual alcohol intake (%) | 568 (32.9) | 479 (31.6) | 548 (33.8) | 550 (34.1) | 400 (32.1) | 369 (31.8) | 418 (35.8) | 394 (32.3) | 452 (34.9) | 468 (33.6) | 483 (33.2) | 448 (31.8) |
| COPD (%) | 85 (4.9) | 87 (5.7) | 76 (4.7) | 88 (5.5) | 61 (4.9) | 66 (5.7) | 60 (5.1) | 54 (4.4) | 57 (4.4) | 65 (4.7) | 67 (4.6) | 67 (4.8) |
| Congestive heart disease (%) | 9 (0.5) | 9 (0.6) | 11 (0.7) | 6 (0.4) | 11 (0.9) | 4 (0.3) | 10 (0.9) | 6 (0.5) | 9 (0.7) | 11 (0.8) | 7 (0.5) | 11 (0.8) |
| Ischemic heart disease (%) | 14 (0.8) | 16 (1.1) | 18 (1.1) | 26 (1.6) | 16 (1.3) | 15 (1.3) | 14 (1.2) | 21 (1.7) | 22 (1.7) | 21 (1.5) | 20 (1.4) | 16 (1.1) |
| Hypertension(%) | 787 (45.6) | 665 (43.8) | 749 (46.2) | 737 (45.7) | 589 (47.2) | 576 (49.6) | 547 (46.8) | 562 (46.0) | 628 (48.5) | 638 (45.8) | 678 (46.7) | 642 (45.6) |
| Dialysis (%) | 9 (0.5) | 14 (0.9) | 15 (0.9) | 13 (0.8) | 14 (1.1) | 13 (1.1) | 9 (0.8) | 12 (1.0) | 6 (0.5) | 10 (0.7) | 14 (1.0) | 11 (0.8) |
| Cerebrovascular disease (%) | 63 (3.7) | 69 (4.5) | 62 (3.8) | 60 (3.7) | 50 (4.0) | 67 (5.8) | 56 (4.8) | 66 (5.4) | 59 (4.6) | 65 (4.7) | 78 (5.4) | 68 (4.8) |
| Steroid (%) | 14 (0.8) | 13 (0.9) | 13 (0.8) | 17 (1.1) | 11 (0.9) | 8 (0.7) | 21 (1.8) | 13 (1.1) | 16 (1.2) | 18 (1.3) | 15 (1.0) | 10 (0.7) |
| Weight loss (%) | 36 (2.1) | 27 (1.8) | 22 (1.4) | 23 (1.4) | 25 (2.0) | 34 (2.9) | 27 (2.3) | 36 (2.9) | 39 (3.0) | 44 (3.2) | 43 (3.0) | 31 (2.2) |
| Bleeding disorder (%) | 232 (13.4) | 196 (12.9) | 213 (13.1) | 224 (13.9) | 170 (13.6) | 185 (15.9) | 161 (13.8) | 200 (16.4) | 224 (17.3) | 208 (14.9) | 215 (14.8) | 186 (13.2) |
| Preoperative transfusion (%) | 11 (0.6) | 20 (1.3) | 20 (1.2) | 16 (1.0) | 19 (1.5) | 20 (1.7) | 16 (1.4) | 21 (1.7) | 14 (1.1) | 14 (1.0) | 21 (1.4) | 16 (1.1) |
| ASA PS (%) |  |  |  |  |  |  |  |  |  |  |  |  |
| ASA1 | 301 (17.4) | 233 (15.4) | 243 (15.0) | 262 (16.2) | 181 (14.5) | 145 (12.5) | 153 (13.1) | 187 (15.3) | 184 (14.2) | 208 (14.9) | 243 (16.7) | 220 (15.6) |
| ASA2 | 1228 (71.1) | 1083 (71.4) | 1186 (73.2) | 1143 (70.8) | 901 (72.3) | 834 (71.8) | 830 (71.1) | 851 (69.7) | 939 (72.6) | 982 (70.5) | 1016 (69.9) | 1003 (71.2) |
| ASA3_5 | 197 (11.4) | 201 (13.2) | 192 (11.8) | 209 (12.9) | 165 (13.2) | 183 (15.7) | 185 (15.8) | 183 (15.0) | 171 (13.2) | 203 (14.6) | 194 (13.4) | 185 (13.1) |
| T factor (%) |  |  |  |  |  |  |  |  |  |  |  |  |
| T0 | 10 (0.6) | 3 (0.2) | 7 (0.4) | 4 (0.2) | 3 (0.2) | 4 (0.3) | 5 (0.4) | 4 (0.3) | 6 (0.5) | 3 (0.2) | 7 (0.5) | 6 (0.4) |
| Tis | 4 (0.2) | 0 (0.0) | 6 (0.4) | 4 (0.2) | 4 (0.3) | 3 (0.3) | 3 (0.3) | 2 (0.2) | 3 (0.2) | 5 (0.4) | 5 (0.3) | 2 (0.1) |
| T1a | 387 (22.4) | 345 (22.7) | 330 (20.4) | 309 (19.1) | 247 (19.8) | 239 (20.6) | 194 (16.6) | 245 (20.1) | 243 (18.8) | 257 (18.4) | 271 (18.7) | 264 (18.8) |
| T1b | 754 (43.7) | 665 (43.8) | 719 (44.4) | 719 (44.5) | 559 (44.8) | 462 (39.8) | 479 (41.0) | 468 (38.3) | 512 (39.6) | 551 (39.6) | 565 (38.9) | 557 (39.6) |
| T2 | 242 (14.0) | 200 (13.2) | 217 (13.4) | 223 (13.8) | 161 (12.9) | 157 (13.5) | 176 (15.1) | 170 (13.9) | 183 (14.1) | 211 (15.1) | 210 (14.5) | 228 (16.2) |
| T3 | 196 (11.4) | 173 (11.4) | 201 (12.4) | 196 (12.1) | 138 (11.1) | 160 (13.8) | 179 (15.3) | 176 (14.4) | 181 (14.0) | 193 (13.9) | 223 (15.3) | 187 (13.3) |
| T4a | 123 (7.1) | 121 (8.0) | 136 (8.4) | 145 (9.0) | 124 (9.9) | 125 (10.8) | 121 (10.4) | 145 (11.9) | 150 (11.6) | 158 (11.3) | 150 (10.3) | 159 (11.3) |
| T4b | 10 (0.6) | 10 (0.7) | 5 (0.3) | 14 (0.9) | 11 (0.9) | 12 (1.0) | 11 (0.9) | 11 (0.9) | 16 (1.2) | 15 (1.1) | 22 (1.5) | 5 (0.4) |
| N factor (%) |  |  |  |  |  |  |  |  |  |  |  |  |
| N0 | 1350 (78.2) | 1178 (77.7) | 1249 (77.1) | 1216 (75.3) | 956 (76.7) | 840 (72.3) | 849 (72.7) | 876 (71.7) | 917 (70.9) | 984 (70.6) | 1068 (73.5) | 1012 (71.9) |
| N1 | 194 (11.2) | 163 (10.7) | 193 (11.9) | 189 (11.7) | 133 (10.7) | 148 (12.7) | 153 (13.1) | 141 (11.5) | 186 (14.4) | 197 (14.1) | 160 (11.0) | 205 (14.6) |
| N2 | 111 (6.4) | 114 (7.5) | 106 (6.5) | 110 (6.8) | 84 (6.7) | 108 (9.3) | 96 (8.2) | 118 (9.7) | 108 (8.3) | 111 (8.0) | 125 (8.6) | 108 (7.7) |
| N3a | 45 (2.6) | 47 (3.1) | 58 (3.6) | 75 (4.6) | 54 (4.3) | 48 (4.1) | 53 (4.5) | 58 (4.8) | 55 (4.3) | 75 (5.4) | 71 (4.9) | 70 (5.0) |
| N3b | 26 (1.5) | 15 (1.0) | 15 (0.9) | 24 (1.5) | 20 (1.6) | 18 (1.5) | 17 (1.5) | 28 (2.3) | 28 (2.2) | 26 (1.9) | 29 (2.0) | 13 (0.9) |
| M1 (%) | 26 (1.5) | 17 (1.1) | 22 (1.4) | 31 (1.9) | 29 (2.3) | 22 (1.9) | 22 (1.9) | 28 (2.3) | 25 (1.9) | 38 (2.7) | 35 (2.4) | 35 (2.5) |

| 2021 | 1 | 2 | 3 | 4 | 5 | 6 | 7 | 8 | 9 | 10 | 11 | 12 |
| --- | --- | --- | --- | --- | --- | --- | --- | --- | --- | --- | --- | --- |
| n | 1508 | 1419 | 1609 | 1544 | 1364 | 1523 | 1326 | 1407 | 1437 | 1434 | 1480 | 1529 |
| age  (median [IQR]) | 73 [66, 78] | 73 [65, 79] | 73 [66, 79] | 72 [65, 79] | 73 [66, 79] | 73 [65, 79] | 73 [65, 79] | 73 [66, 79] | 73 [66, 79] | 73 [67, 79] | 73 [67, 79] | 73 [66, 79] |
| category of age (%) |  |  |  |  |  |  |  |  |  |  |  |  |
| <65 | 309 (20.5) | 330 (23.3) | 350 (21.8) | 380 (24.6) | 300 (22.0) | 351 (23.0) | 310 (23.4) | 309 (22.0) | 314 (21.9) | 290 (20.2) | 289 (19.5) | 328 (21.5) |
| 65-75 | 636 (42.2) | 524 (36.9) | 625 (38.8) | 590 (38.2) | 524 (38.4) | 588 (38.6) | 527 (39.7) | 564 (40.1) | 572 (39.8) | 592 (41.3) | 621 (42.0) | 632 (41.3) |
| 75< | 563 (37.3) | 565 (39.8) | 634 (39.4) | 574 (37.2) | 540 (39.6) | 584 (38.3) | 489 (36.9) | 534 (38.0) | 551 (38.3) | 552 (38.5) | 570 (38.5) | 569 (37.2) |
| male (%) | 1028 (68.2) | 948 (66.8) | 1042 (64.8) | 976 (63.2) | 906 (66.4) | 1001 (65.7) | 897 (67.6) | 957 (68.0) | 974 (67.8) | 970 (67.6) | 975 (65.9) | 995 (65.1) |
| BMI≥25 (%) | 380 (25.2) | 336 (23.7) | 410 (25.5) | 374 (24.2) | 321 (23.5) | 335 (22.0) | 305 (23.0) | 313 (22.2) | 316 (22.0) | 334 (23.3) | 350 (23.6) | 372 (24.3) |
| Preoperative chemotherapy (%) | 41 (2.7) | 31 (2.2) | 33 (2.1) | 32 (2.1) | 42 (3.1) | 42 (2.8) | 41 (3.1) | 43 (3.1) | 35 (2.4) | 46 (3.2) | 32 (2.2) | 40 (2.6) |
| DM (%) | 289 (19.2) | 234 (16.5) | 324 (20.1) | 276 (17.9) | 257 (18.8) | 294 (19.3) | 262 (19.8) | 272 (19.3) | 271 (18.9) | 261 (18.2) | 299 (20.2) | 266 (17.4) |
| Brinkman index (%) |  |  |  |  |  |  |  |  |  |  |  |  |
| 0 | 666 (44.2) | 664 (46.8) | 770 (47.9) | 737 (47.7) | 619 (45.4) | 673 (44.2) | 590 (44.5) | 656 (46.6) | 668 (46.5) | 681 (47.5) | 685 (46.3) | 715 (46.8) |
| <400 | 252 (16.7) | 228 (16.1) | 254 (15.8) | 236 (15.3) | 217 (15.9) | 246 (16.2) | 229 (17.3) | 202 (14.4) | 233 (16.2) | 206 (14.4) | 241 (16.3) | 242 (15.8) |
| ≥400 | 590 (39.1) | 527 (37.1) | 585 (36.4) | 571 (37.0) | 528 (38.7) | 604 (39.7) | 507 (38.2) | 549 (39.0) | 536 (37.3) | 547 (38.1) | 554 (37.4) | 572 (37.4) |
| Habitual alcohol intake (%) | 512 (34.0) | 479 (33.8) | 543 (33.7) | 488 (31.6) | 482 (35.3) | 527 (34.6) | 448 (33.8) | 454 (32.3) | 503 (35.0) | 490 (34.2) | 484 (32.7) | 498 (32.6) |
| COPD (%) | 70 (4.6) | 76 (5.4) | 68 (4.2) | 74 (4.8) | 76 (5.6) | 72 (4.7) | 61 (4.6) | 77 (5.5) | 87 (6.1) | 70 (4.9) | 60 (4.1) | 79 (5.2) |
| Congestive heart disease (%) | 4 (0.3) | 18 (1.3) | 14 (0.9) | 10 (0.6) | 13 (1.0) | 12 (0.8) | 14 (1.1) | 11 (0.8) | 11 (0.8) | 12 (0.8) | 11 (0.7) | 7 (0.5) |
| Ischemic heart disease (%) | 27 (1.8) | 24 (1.7) | 26 (1.6) | 28 (1.8) | 18 (1.3) | 30 (2.0) | 21 (1.6) | 19 (1.4) | 14 (1.0) | 13 (0.9) | 18 (1.2) | 20 (1.3) |
| Hypertension(%) | 692 (45.9) | 663 (46.7) | 795 (49.4) | 699 (45.3) | 683 (50.1) | 711 (46.7) | 638 (48.1) | 664 (47.2) | 687 (47.8) | 712 (49.7) | 723 (48.9) | 705 (46.1) |
| Dialysis (%) | 7 (0.5) | 10 (0.7) | 20 (1.2) | 5 (0.3) | 17 (1.2) | 16 (1.1) | 13 (1.0) | 11 (0.8) | 8 (0.6) | 10 (0.7) | 13 (0.9) | 21 (1.4) |
| Cerebrovascular disease (%) | 71 (4.7) | 60 (4.2) | 71 (4.4) | 60 (3.9) | 57 (4.2) | 71 (4.7) | 61 (4.6) | 77 (5.5) | 62 (4.3) | 76 (5.3) | 68 (4.6) | 61 (4.0) |
| Steroid (%) | 14 (0.9) | 17 (1.2) | 18 (1.1) | 17 (1.1) | 14 (1.0) | 20 (1.3) | 19 (1.4) | 15 (1.1) | 10 (0.7) | 17 (1.2) | 20 (1.4) | 19 (1.2) |
| Weight loss (%) | 33 (2.2) | 27 (1.9) | 41 (2.5) | 38 (2.5) | 25 (1.8) | 41 (2.7) | 43 (3.2) | 37 (2.6) | 38 (2.6) | 39 (2.7) | 38 (2.6) | 34 (2.2) |
| Bleeding disorder (%) | 228 (15.1) | 214 (15.1) | 253 (15.7) | 222 (14.4) | 205 (15.0) | 233 (15.3) | 217 (16.4) | 219 (15.6) | 220 (15.3) | 216 (15.1) | 228 (15.4) | 195 (12.8) |
| Preoperative transfusion (%) | 13 (0.9) | 17 (1.2) | 30 (1.9) | 22 (1.4) | 17 (1.2) | 36 (2.4) | 20 (1.5) | 21 (1.5) | 22 (1.5) | 19 (1.3) | 18 (1.2) | 30 (2.0) |
| ASA PS (%) |  |  |  |  |  |  |  |  |  |  |  |  |
| ASA1 | 216 (14.3) | 213 (15.0) | 225 (14.0) | 236 (15.3) | 150 (11.0) | 192 (12.6) | 169 (12.7) | 174 (12.4) | 163 (11.3) | 190 (13.2) | 187 (12.6) | 195 (12.8) |
| ASA2 | 1112 (73.7) | 1015 (71.5) | 1134 (70.5) | 1111 (72.0) | 1002 (73.5) | 1109 (72.8) | 966 (72.9) | 1030 (73.2) | 1059 (73.7) | 1057 (73.7) | 1085 (73.3) | 1127 (73.7) |
| ASA3_5 | 180 (11.9) | 191 (13.5) | 250 (15.5) | 197 (12.8) | 212 (15.5) | 222 (14.6) | 191 (14.4) | 203 (14.4) | 215 (15.0) | 187 (13.0) | 208 (14.1) | 207 (13.5) |
| T factor (%) |  |  |  |  |  |  |  |  |  |  |  |  |
| T0 | 6 (0.4) | 3 (0.2) | 4 (0.2) | 5 (0.3) | 9 (0.7) | 3 (0.2) | 8 (0.6) | 5 (0.4) | 1 (0.1) | 11 (0.8) | 7 (0.5) | 2 (0.1) |
| Tis | 3 (0.2) | 5 (0.4) | 5 (0.3) | 1 (0.1) | 2 (0.1) | 3 (0.2) | 4 (0.3) | 2 (0.1) | 4 (0.3) | 2 (0.1) | 3 (0.2) | 2 (0.1) |
| T1a | 330 (21.9) | 301 (21.2) | 315 (19.6) | 311 (20.1) | 233 (17.1) | 275 (18.1) | 224 (16.9) | 241 (17.1) | 268 (18.6) | 236 (16.5) | 265 (17.9) | 280 (18.3) |
| T1b | 639 (42.4) | 590 (41.6) | 647 (40.2) | 604 (39.1) | 571 (41.9) | 578 (38.0) | 520 (39.2) | 572 (40.7) | 593 (41.3) | 595 (41.5) | 574 (38.8) | 629 (41.1) |
| T2 | 210 (13.9) | 209 (14.7) | 210 (13.1) | 226 (14.6) | 216 (15.8) | 231 (15.2) | 199 (15.0) | 199 (14.1) | 207 (14.4) | 192 (13.4) | 226 (15.3) | 217 (14.2) |
| T3 | 184 (12.2) | 188 (13.2) | 226 (14.0) | 219 (14.2) | 186 (13.6) | 234 (15.4) | 206 (15.5) | 193 (13.7) | 191 (13.3) | 214 (14.9) | 233 (15.7) | 227 (14.8) |
| T4a | 126 (8.4) | 109 (7.7) | 186 (11.6) | 166 (10.8) | 134 (9.8) | 190 (12.5) | 151 (11.4) | 180 (12.8) | 155 (10.8) | 171 (11.9) | 161 (10.9) | 159 (10.4) |
| T4b | 10 (0.7) | 14 (1.0) | 16 (1.0) | 12 (0.8) | 13 (1.0) | 9 (0.6) | 14 (1.1) | 15 (1.1) | 18 (1.3) | 13 (0.9) | 11 (0.7) | 13 (0.9) |
| N factor (%) |  |  |  |  |  |  |  |  |  |  |  |  |
| N0 | 1162 (77.1) | 1087 (76.6) | 1188 (73.8) | 1133 (73.4) | 1002 (73.5) | 1070 (70.3) | 939 (70.8) | 1017 (72.3) | 1062 (73.9) | 1005 (70.1) | 1067 (72.1) | 1133 (74.1) |
| N1 | 172 (11.4) | 167 (11.8) | 195 (12.1) | 169 (10.9) | 163 (12.0) | 202 (13.3) | 192 (14.5) | 198 (14.1) | 174 (12.1) | 200 (13.9) | 185 (12.5) | 184 (12.0) |
| N2 | 110 (7.3) | 107 (7.5) | 127 (7.9) | 134 (8.7) | 121 (8.9) | 149 (9.8) | 112 (8.4) | 102 (7.2) | 112 (7.8) | 129 (9.0) | 135 (9.1) | 118 (7.7) |
| N3a | 44 (2.9) | 44 (3.1) | 63 (3.9) | 75 (4.9) | 60 (4.4) | 74 (4.9) | 62 (4.7) | 60 (4.3) | 72 (5.0) | 72 (5.0) | 70 (4.7) | 68 (4.4) |
| N3b | 20 (1.3) | 14 (1.0) | 36 (2.2) | 33 (2.1) | 18 (1.3) | 28 (1.8) | 21 (1.6) | 30 (2.1) | 17 (1.2) | 28 (2.0) | 23 (1.6) | 26 (1.7) |
| M1 (%) | 28 (1.9) | 27 (1.9) | 36 (2.2) | 36 (2.3) | 26 (1.9) | 39 (2.6) | 23 (1.7) | 29 (2.1) | 31 (2.2) | 31 (2.2) | 27 (1.8) | 33 (2.2) |

Supplement 1b Background of patients with LLAR

| 2018 | 1 | 2 | 3 | 4 | 5 | 6 | 7 | 8 | 9 | 10 | 11 | 12 |
| --- | --- | --- | --- | --- | --- | --- | --- | --- | --- | --- | --- | --- |
| n | 1271 | 1233 | 1295 | 1213 | 1293 | 1321 | 1276 | 1324 | 1156 | 1428 | 1277 | 1233 |
| age (median [IQR]) | 68  [61, 74] | 68  [61, 74] | 68  [60, 74] | 69  [61, 75] | 69  [61, 75] | 69  [61, 76] | 69  [61, 76] | 69  [61, 74] | 68  [59, 75] | 69  [60, 75] | 68  [60, 75] | 68  [60, 75] |
| category of age (%) |  |  |  |  |  |  |  |  |  |  |  |  |
| <65 | 472 (37.1) | 447 (36.3) | 482 (37.2) | 420 (34.6) | 428 (33.1) | 431 (32.6) | 448 (35.1) | 469 (35.4) | 451 (39.0) | 499 (34.9) | 477 (37.4) | 459 (37.2) |
| 65-75 | 529 (41.6) | 524 (42.5) | 541 (41.8) | 495 (40.8) | 568 (43.9) | 539 (40.8) | 508 (39.8) | 564 (42.6) | 432 (37.4) | 586 (41.0) | 491 (38.4) | 485 (39.3) |
| 75< | 270 (21.2) | 262 (21.2) | 272 (21.0) | 298 (24.6) | 297 (23.0) | 351 (26.6) | 320 (25.1) | 291 (22.0) | 273 (23.6) | 343 (24.0) | 309 (24.2) | 289 (23.4) |
| male (%) | 862 (67.8) | 802 (65.0) | 853 (65.9) | 823 (67.8) | 845 (65.4) | 869 (65.8) | 814 (63.8) | 881 (66.5) | 763 (66.0) | 1001 (70.1) | 836 (65.5) | 760 (61.6) |
| BMI≥25 (%) | 352 (27.7) | 338 (27.4) | 341 (26.3) | 302 (24.9) | 291 (22.5) | 321 (24.3) | 317 (24.8) | 279 (21.1) | 283 (24.5) | 343 (24.0) | 314 (24.6) | 293 (23.8) |
| Preoperative chemotherapy (%) | 124 (9.8) | 95 (7.7) | 100 (7.7) | 105 (8.7) | 117 (9.0) | 116 (8.8) | 121 (9.5) | 105 (7.9) | 106 (9.2) | 122 (8.5) | 99 (7.8) | 84 (6.8) |
| DM (%) | 198 (15.6) | 223 (18.1) | 208 (16.1) | 224 (18.5) | 234 (18.1) | 252 (19.1) | 212 (16.6) | 211 (15.9) | 196 (17.0) | 225 (15.8) | 201 (15.7) | 195 (15.8) |
| Brinkman index (%) |  |  |  |  |  |  |  |  |  |  |  |  |
| 0 | 660 (51.9) | 646 (52.4) | 662 (51.1) | 619 (51.0) | 648 (50.1) | 656 (49.7) | 649 (50.9) | 651 (49.2) | 579 (50.1) | 686 (48.0) | 647 (50.7) | 652 (52.9) |
| <400 | 192 (15.1) | 194 (15.7) | 200 (15.4) | 181 (14.9) | 186 (14.4) | 218 (16.5) | 209 (16.4) | 212 (16.0) | 168 (14.5) | 254 (17.8) | 203 (15.9) | 202 (16.4) |
| ≥400 | 419 (33.0) | 393 (31.9) | 433 (33.4) | 413 (34.0) | 459 (35.5) | 447 (33.8) | 418 (32.8) | 461 (34.8) | 409 (35.4) | 488 (34.2) | 427 (33.4) | 379 (30.7) |
| Habitual alcohol intake (%) | 429 (33.8) | 410 (33.3) | 442 (34.1) | 400 (33.0) | 432 (33.4) | 435 (32.9) | 419 (32.8) | 456 (34.4) | 410 (35.5) | 526 (36.8) | 418 (32.7) | 394 (32.0) |
| COPD (%) | 52 (4.1) | 35 (2.8) | 37 (2.9) | 53 (4.4) | 42 (3.2) | 51 (3.9) | 41 (3.2) | 44 (3.3) | 28 (2.4) | 53 (3.7) | 39 (3.1) | 43 (3.5) |
| Congestive heart disease (%) | 7 (0.6) | 10 (0.8) | 4 (0.3) | 10 (0.8) | 4 (0.3) | 5 (0.4) | 3 (0.2) | 3 (0.2) | 3 (0.3) | 11 (0.8) | 5 (0.4) | 2 (0.2) |
| Ischemic heart disease (%) | 15 (1.2) | 16 (1.3) | 14 (1.1) | 13 (1.1) | 13 (1.0) | 22 (1.7) | 13 (1.0) | 16 (1.2) | 15 (1.3) | 20 (1.4) | 11 (0.9) | 7 (0.6) |
| Hypertension(%) | 509 (40.0) | 481 (39.0) | 499 (38.5) | 477 (39.3) | 506 (39.1) | 533 (40.3) | 525 (41.1) | 474 (35.8) | 436 (37.7) | 567 (39.7) | 484 (37.9) | 465 (37.7) |
| Dialysis (%) | 6 (0.5) | 12 (1.0) | 10 (0.8) | 4 (0.3) | 7 (0.5) | 8 (0.6) | 6 (0.5) | 6 (0.5) | 6 (0.5) | 5 (0.4) | 9 (0.7) | 5 (0.4) |
| Cerebrovascular disease (%) | 41 (3.2) | 37 (3.0) | 47 (3.6) | 38 (3.1) | 47 (3.6) | 41 (3.1) | 44 (3.4) | 45 (3.4) | 45 (3.9) | 53 (3.7) | 48 (3.8) | 36 (2.9) |
| Steroid (%) | 9 (0.7) | 9 (0.7) | 9 (0.7) | 8 (0.7) | 11 (0.9) | 13 (1.0) | 8 (0.6) | 4 (0.3) | 9 (0.8) | 13 (0.9) | 6 (0.5) | 11 (0.9) |
| Weight loss (%) | 23 (1.8) | 20 (1.6) | 25 (1.9) | 14 (1.2) | 32 (2.5) | 36 (2.7) | 22 (1.7) | 26 (2.0) | 12 (1.0) | 29 (2.0) | 30 (2.3) | 25 (2.0) |
| Bleeding disorder (%) | 123 (9.7) | 101 (8.2) | 101 (7.8) | 111 (9.2) | 121 (9.4) | 131 (9.9) | 116 (9.1) | 127 (9.6) | 117 (10.1) | 141 (9.9) | 114 (8.9) | 121 (9.8) |
| Preoperative transfusion (%) | 6 (0.5) | 12 (1.0) | 15 (1.2) | 13 (1.1) | 8 (0.6) | 21 (1.6) | 12 (0.9) | 4 (0.3) | 10 (0.9) | 16 (1.1) | 6 (0.5) | 12 (1.0) |
| ASA PS (%) |  |  |  |  |  |  |  |  |  |  |  |  |
| ASA1 | 293 (23.1) | 276 (22.4) | 284 (21.9) | 266 (21.9) | 274 (21.2) | 260 (19.7) | 253 (19.8) | 316 (23.9) | 260 (22.5) | 309 (21.6) | 283 (22.2) | 271 (22.0) |
| ASA2 | 863 (67.9) | 835 (67.7) | 856 (66.1) | 837 (69.0) | 889 (68.8) | 910 (68.9) | 885 (69.4) | 876 (66.2) | 764 (66.1) | 955 (66.9) | 856 (67.0) | 845 (68.5) |
| ASA3_5 | 115 (9.0) | 122 (9.9) | 155 (12.0) | 110 (9.1) | 130 (10.1) | 151 (11.4) | 138 (10.8) | 132 (10.0) | 132 (11.4) | 164 (11.5) | 138 (10.8) | 117 (9.5) |
| T factor (%) |  |  |  |  |  |  |  |  |  |  |  |  |
| T0 | 4 (0.3) | 7 (0.6) | 4 (0.3) | 8 (0.7) | 7 (0.5) | 9 (0.7) | 8 (0.6) | 13 (1.0) | 8 (0.7) | 12 (0.8) | 7 (0.5) | 6 (0.5) |
| Tis | 34 (2.7) | 19 (1.5) | 23 (1.8) | 24 (2.0) | 22 (1.7) | 25 (1.9) | 19 (1.5) | 24 (1.8) | 17 (1.5) | 22 (1.5) | 16 (1.3) | 17 (1.4) |
| T1 | 262 (20.6) | 250 (20.3) | 219 (16.9) | 205 (16.9) | 206 (15.9) | 210 (15.9) | 186 (14.6) | 205 (15.5) | 181 (15.7) | 237 (16.6) | 217 (17.0) | 207 (16.8) |
| T2 | 263 (20.7) | 247 (20.0) | 253 (19.5) | 242 (20.0) | 237 (18.3) | 234 (17.7) | 226 (17.7) | 231 (17.4) | 221 (19.1) | 273 (19.1) | 243 (19.0) | 217 (17.6) |
| T3 | 568 (44.7) | 553 (44.8) | 628 (48.5) | 572 (47.2) | 627 (48.5) | 653 (49.4) | 632 (49.5) | 640 (48.3) | 571 (49.4) | 683 (47.8) | 600 (47.0) | 601 (48.7) |
| T4a | 110 (8.7) | 129 (10.5) | 135 (10.4) | 141 (11.6) | 165 (12.8) | 155 (11.7) | 167 (13.1) | 169 (12.8) | 129 (11.2) | 149 (10.4) | 158 (12.4) | 152 (12.3) |
| T4b | 30 (2.4) | 28 (2.3) | 33 (2.5) | 21 (1.7) | 29 (2.2) | 35 (2.6) | 38 (3.0) | 42 (3.2) | 29 (2.5) | 52 (3.6) | 36 (2.8) | 33 (2.7) |
| N factor (%) |  |  |  |  |  |  |  |  |  |  |  |  |
| N0 | 810 (63.7) | 775 (62.9) | 776 (59.9) | 746 (61.5) | 759 (58.7) | 789 (59.7) | 753 (59.0) | 782 (59.1) | 695 (60.1) | 851 (59.6) | 771 (60.4) | 734 (59.5) |
| N1a | 195 (15.3) | 171 (13.9) | 191 (14.7) | 165 (13.6) | 202 (15.6) | 202 (15.3) | 199 (15.6) | 192 (14.5) | 167 (14.4) | 195 (13.7) | 170 (13.3) | 174 (14.1) |
| N1b | 110 (8.7) | 138 (11.2) | 155 (12.0) | 140 (11.5) | 165 (12.8) | 141 (10.7) | 146 (11.4) | 144 (10.9) | 123 (10.6) | 170 (11.9) | 155 (12.1) | 155 (12.6) |
| N1c | 3 (0.2) | 5 (0.4) | 5 (0.4) | 6 (0.5) | 4 (0.3) | 5 (0.4) | 4 (0.3) | 4 (0.3) | 4 (0.3) | 5 (0.4) | 2 (0.2) | 3 (0.2) |
| N2a | 97 (7.6) | 92 (7.5) | 99 (7.6) | 104 (8.6) | 101 (7.8) | 132 (10.0) | 113 (8.9) | 127 (9.6) | 106 (9.2) | 116 (8.1) | 105 (8.2) | 101 (8.2) |
| N2b | 56 (4.4) | 52 (4.2) | 69 (5.3) | 52 (4.3) | 62 (4.8) | 52 (3.9) | 61 (4.8) | 75 (5.7) | 61 (5.3) | 91 (6.4) | 74 (5.8) | 66 (5.4) |
| M factor (%) |  |  |  |  |  |  |  |  |  |  |  |  |
| M0 | 1190 (93.6) | 1144 (92.8) | 1184 (91.4) | 1125 (92.7) | 1176 (91.0) | 1215 (92.0) | 1166 (91.4) | 1206 (91.1) | 1054 (91.2) | 1317 (92.2) | 1189 (93.1) | 1142 (92.6) |
| M1a | 64 (5.0) | 68 (5.5) | 87 (6.7) | 71 (5.9) | 88 (6.8) | 85 (6.4) | 86 (6.7) | 83 (6.3) | 76 (6.6) | 92 (6.4) | 72 (5.6) | 73 (5.9) |
| M1b | 13 (1.0) | 18 (1.5) | 17 (1.3) | 13 (1.1) | 21 (1.6) | 13 (1.0) | 18 (1.4) | 18 (1.4) | 14 (1.2) | 16 (1.1) | 9 (0.7) | 14 (1.1) |
| M1c | 4 (0.3) | 3 (0.2) | 7 (0.5) | 4 (0.3) | 8 (0.6) | 8 (0.6) | 6 (0.5) | 17 (1.3) | 12 (1.0) | 3 (0.2) | 7 (0.5) | 4 (0.3) |
|  |  |  |  |  |  |  |  |  |  |  |  |  |
| 2019 | 1 | 2 | 3 | 4 | 5 | 6 | 7 | 8 | 9 | 10 | 11 | 12 |
| n | 1417 | 1342 | 1346 | 1434 | 1324 | 1303 | 1439 | 1334 | 1291 | 1454 | 1309 | 1432 |
| age (median [IQR]) | 69  [60, 75] | 68  [60, 75] | 69  [61, 74] | 68  [60, 75] | 69  [61, 75] | 69  [61, 75] | 69  [61, 75] | 69  [60, 75] | 69  [61, 76] | 68  [60, 75] | 69  [61, 76] | 69  [61, 75] |
| category of age (%) |  |  |  |  |  |  |  |  |  |  |  |  |
| <65 | 494 (34.9) | 470 (35.0) | 459 (34.1) | 508 (35.4) | 435 (32.9) | 441 (33.8) | 484 (33.6) | 477 (35.8) | 433 (33.5) | 538 (37.0) | 448 (34.2) | 494 (34.5) |
| 65-75 | 581 (41.0) | 573 (42.7) | 592 (44.0) | 608 (42.4) | 561 (42.4) | 545 (41.8) | 597 (41.5) | 532 (39.9) | 523 (40.5) | 570 (39.2) | 525 (40.1) | 581 (40.6) |
| 75< | 342 (24.1) | 299 (22.3) | 295 (21.9) | 318 (22.2) | 328 (24.8) | 317 (24.3) | 358 (24.9) | 325 (24.4) | 335 (25.9) | 346 (23.8) | 336 (25.7) | 357 (24.9) |
| male (%) | 920 (64.9) | 889 (66.2) | 893 (66.3) | 924 (64.4) | 837 (63.2) | 837 (64.2) | 942 (65.5) | 858 (64.3) | 860 (66.6) | 972 (66.9) | 864 (66.0) | 944 (65.9) |
| BMI≥25 (%) | 350 (24.7) | 371 (27.6) | 320 (23.8) | 374 (26.1) | 344 (26.0) | 340 (26.1) | 338 (23.5) | 332 (24.9) | 284 (22.0) | 355 (24.4) | 297 (22.7) | 342 (23.9) |
| Preoperative chemotherapy (%) | 135 (9.5) | 120 (8.9) | 99 (7.4) | 111 (7.7) | 131 (9.9) | 103 (7.9) | 119 (8.3) | 98 (7.3) | 109 (8.4) | 135 (9.3) | 121 (9.2) | 110 (7.7) |
| DM (%) | 231 (16.3) | 268 (20.0) | 262 (19.5) | 273 (19.0) | 244 (18.4) | 218 (16.7) | 275 (19.1) | 231 (17.3) | 225 (17.4) | 241 (16.6) | 223 (17.0) | 255 (17.8) |
| Brinkman index (%) |  |  |  |  |  |  |  |  |  |  |  |  |
| 0 | 711 (50.2) | 665 (49.6) | 632 (47.0) | 739 (51.5) | 643 (48.6) | 652 (50.0) | 740 (51.4) | 637 (47.8) | 653 (50.6) | 665 (45.7) | 626 (47.8) | 677 (47.3) |
| <400 | 226 (15.9) | 223 (16.6) | 240 (17.8) | 207 (14.4) | 221 (16.7) | 220 (16.9) | 220 (15.3) | 213 (16.0) | 212 (16.4) | 236 (16.2) | 207 (15.8) | 243 (17.0) |
| ≥400 | 480 (33.9) | 454 (33.8) | 474 (35.2) | 488 (34.0) | 460 (34.7) | 431 (33.1) | 479 (33.3) | 484 (36.3) | 426 (33.0) | 553 (38.0) | 476 (36.4) | 512 (35.8) |
| Habitual alcohol intake (%) | 467 (33.0) | 465 (34.6) | 467 (34.7) | 458 (31.9) | 431 (32.6) | 440 (33.8) | 469 (32.6) | 467 (35.0) | 439 (34.0) | 537 (36.9) | 432 (33.0) | 486 (33.9) |
| COPD (%) | 55 (3.9) | 39 (2.9) | 55 (4.1) | 36 (2.5) | 44 (3.3) | 44 (3.4) | 49 (3.4) | 62 (4.6) | 53 (4.1) | 54 (3.7) | 48 (3.7) | 44 (3.1) |
| Congestive heart disease (%) | 12 (0.8) | 6 (0.4) | 7 (0.5) | 1 (0.1) | 11 (0.8) | 3 (0.2) | 6 (0.4) | 4 (0.3) | 3 (0.2) | 6 (0.4) | 6 (0.5) | 7 (0.5) |
| Ischemic heart disease (%) | 12 (0.8) | 15 (1.1) | 18 (1.3) | 18 (1.3) | 23 (1.7) | 14 (1.1) | 23 (1.6) | 12 (0.9) | 12 (0.9) | 12 (0.8) | 13 (1.0) | 10 (0.7) |
| Hypertension(%) | 558 (39.4) | 552 (41.1) | 520 (38.6) | 570 (39.7) | 537 (40.6) | 576 (44.2) | 582 (40.4) | 503 (37.7) | 516 (40.0) | 567 (39.0) | 514 (39.3) | 559 (39.0) |
| Dialysis (%) | 5 (0.4) | 7 (0.5) | 4 (0.3) | 6 (0.4) | 9 (0.7) | 8 (0.6) | 7 (0.5) | 10 (0.7) | 5 (0.4) | 3 (0.2) | 2 (0.2) | 6 (0.4) |
| Cerebrovascular disease (%) | 41 (2.9) | 42 (3.1) | 63 (4.7) | 51 (3.6) | 49 (3.7) | 46 (3.5) | 47 (3.3) | 42 (3.1) | 44 (3.4) | 51 (3.5) | 47 (3.6) | 52 (3.6) |
| Steroid (%) | 14 (1.0) | 11 (0.8) | 11 (0.8) | 17 (1.2) | 10 (0.8) | 9 (0.7) | 8 (0.6) | 15 (1.1) | 8 (0.6) | 9 (0.6) | 3 (0.2) | 6 (0.4) |
| Weight loss (%) | 13 (0.9) | 22 (1.6) | 20 (1.5) | 15 (1.0) | 26 (2.0) | 22 (1.7) | 33 (2.3) | 23 (1.7) | 24 (1.9) | 35 (2.4) | 19 (1.5) | 21 (1.5) |
| Bleeding disorder (%) | 132 (9.3) | 136 (10.1) | 147 (10.9) | 144 (10.0) | 125 (9.4) | 142 (10.9) | 144 (10.0) | 129 (9.7) | 124 (9.6) | 122 (8.4) | 123 (9.4) | 145 (10.1) |
| Preoperative transfusion (%) | 12 (0.8) | 10 (0.7) | 9 (0.7) | 12 (0.8) | 12 (0.9) | 6 (0.5) | 14 (1.0) | 12 (0.9) | 12 (0.9) | 13 (0.9) | 9 (0.7) | 12 (0.8) |
| ASA PS (%) |  |  |  |  |  |  |  |  |  |  |  |  |
| ASA1 | 306 (21.6) | 268 (20.0) | 269 (20.0) | 288 (20.1) | 249 (18.8) | 227 (17.4) | 277 (19.2) | 274 (20.5) | 232 (18.0) | 270 (18.6) | 228 (17.4) | 273 (19.1) |
| ASA2 | 979 (69.1) | 930 (69.3) | 924 (68.6) | 995 (69.4) | 910 (68.7) | 930 (71.4) | 1003 (69.7) | 912 (68.4) | 916 (71.0) | 1039 (71.5) | 947 (72.3) | 998 (69.7) |
| ASA3_5 | 132 (9.3) | 144 (10.7) | 153 (11.4) | 151 (10.5) | 165 (12.5) | 146 (11.2) | 159 (11.0) | 148 (11.1) | 143 (11.1) | 145 (10.0) | 134 (10.2) | 161 (11.2) |
| T factor (%) |  |  |  |  |  |  |  |  |  |  |  |  |
| T0 | 10 (0.7) | 2 (0.1) | 3 (0.2) | 6 (0.4) | 6 (0.5) | 6 (0.5) | 8 (0.6) | 4 (0.3) | 5 (0.4) | 7 (0.5) | 7 (0.5) | 5 (0.3) |
| Tis | 17 (1.2) | 22 (1.6) | 20 (1.5) | 26 (1.8) | 22 (1.7) | 18 (1.4) | 24 (1.7) | 28 (2.1) | 18 (1.4) | 20 (1.4) | 16 (1.2) | 18 (1.3) |
| T1 | 243 (17.1) | 235 (17.5) | 243 (18.1) | 228 (15.9) | 218 (16.5) | 210 (16.1) | 217 (15.1) | 217 (16.3) | 200 (15.5) | 243 (16.7) | 227 (17.3) | 244 (17.0) |
| T2 | 306 (21.6) | 250 (18.6) | 249 (18.5) | 280 (19.5) | 243 (18.4) | 247 (19.0) | 239 (16.6) | 219 (16.4) | 243 (18.8) | 278 (19.1) | 244 (18.6) | 259 (18.1) |
| T3 | 647 (45.7) | 647 (48.2) | 616 (45.8) | 673 (46.9) | 643 (48.6) | 632 (48.5) | 716 (49.8) | 678 (50.8) | 625 (48.4) | 715 (49.2) | 614 (46.9) | 692 (48.3) |
| T4a | 165 (11.6) | 157 (11.7) | 172 (12.8) | 184 (12.8) | 152 (11.5) | 151 (11.6) | 195 (13.6) | 154 (11.5) | 172 (13.3) | 149 (10.2) | 161 (12.3) | 164 (11.5) |
| T4b | 29 (2.0) | 29 (2.2) | 43 (3.2) | 37 (2.6) | 40 (3.0) | 39 (3.0) | 40 (2.8) | 34 (2.5) | 28 (2.2) | 42 (2.9) | 40 (3.1) | 50 (3.5) |
| N factor (%) |  |  |  |  |  |  |  |  |  |  |  |  |
| N0 | 893 (63.0) | 789 (58.8) | 795 (59.1) | 837 (58.4) | 768 (58.0) | 769 (59.0) | 842 (58.5) | 785 (58.8) | 757 (58.6) | 845 (58.1) | 778 (59.4) | 874 (61.0) |
| N1a | 171 (12.1) | 201 (15.0) | 180 (13.4) | 200 (13.9) | 204 (15.4) | 190 (14.6) | 198 (13.8) | 182 (13.6) | 189 (14.6) | 198 (13.6) | 174 (13.3) | 177 (12.4) |
| N1b | 175 (12.4) | 179 (13.3) | 146 (10.8) | 188 (13.1) | 183 (13.8) | 155 (11.9) | 202 (14.0) | 174 (13.0) | 182 (14.1) | 202 (13.9) | 170 (13.0) | 180 (12.6) |
| N1c | 1 (0.1) | 1 (0.1) | 2 (0.1) | 4 (0.3) | 1 (0.1) | 2 (0.2) | 0 (0.0) | 4 (0.3) | 2 (0.2) | 3 (0.2) | 2 (0.2) | 3 (0.2) |
| N2a | 112 (7.9) | 105 (7.8) | 143 (10.6) | 124 (8.6) | 97 (7.3) | 110 (8.4) | 105 (7.3) | 100 (7.5) | 95 (7.4) | 126 (8.7) | 106 (8.1) | 116 (8.1) |
| N2b | 65 (4.6) | 67 (5.0) | 80 (5.9) | 81 (5.6) | 71 (5.4) | 77 (5.9) | 92 (6.4) | 89 (6.7) | 66 (5.1) | 80 (5.5) | 79 (6.0) | 82 (5.7) |
| M factor (%) |  |  |  |  |  |  |  |  |  |  |  |  |
| M0 | 1316 (92.9) | 1228 (91.5) | 1229 (91.3) | 1303 (90.9) | 1225 (92.5) | 1186 (91.0) | 1325 (92.1) | 1221 (91.5) | 1191 (92.3) | 1338 (92.0) | 1195 (91.3) | 1311 (91.6) |
| M1a | 83 (5.9) | 88 (6.6) | 89 (6.6) | 100 (7.0) | 75 (5.7) | 82 (6.3) | 86 (6.0) | 84 (6.3) | 76 (5.9) | 94 (6.5) | 84 (6.4) | 88 (6.1) |
| M1b | 9 (0.6) | 16 (1.2) | 19 (1.4) | 16 (1.1) | 15 (1.1) | 20 (1.5) | 22 (1.5) | 12 (0.9) | 19 (1.5) | 10 (0.7) | 22 (1.7) | 20 (1.4) |
| M1c | 9 (0.6) | 10 (0.7) | 9 (0.7) | 15 (1.0) | 9 (0.7) | 15 (1.2) | 6 (0.4) | 17 (1.3) | 5 (0.4) | 12 (0.8) | 8 (0.6) | 13 (0.9) |
|  |  |  |  |  |  |  |  |  |  |  |  |  |
| 2020 | 1 | 2 | 3 | 4 | 5 | 6 | 7 | 8 | 9 | 10 | 11 | 12 |
| n | 1387 | 1272 | 1438 | 1483 | 1140 | 1258 | 1262 | 1123 | 1255 | 1427 | 1367 | 1364 |
| age (median [IQR]) | 68  [60, 75] | 69  [60, 75] | 69  [60, 75] | 68  [59, 75] | 68  [60, 75] | 70  [61, 76] | 70  [62, 76] | 70  [61, 77] | 69  [60, 75] | 68  [60, 75] | 69  [60, 76] | 69  [61, 76] |
| category of age (%) |  |  |  |  |  |  |  |  |  |  |  |  |
| <65 | 501 (36.1) | 460 (36.2) | 537 (37.3) | 559 (37.7) | 427 (37.5) | 409 (32.5) | 401 (31.8) | 378 (33.7) | 460 (36.7) | 528 (37.0) | 499 (36.5) | 463 (33.9) |
| 65-75 | 569 (41.0) | 517 (40.6) | 560 (38.9) | 586 (39.5) | 462 (40.5) | 491 (39.0) | 541 (42.9) | 434 (38.6) | 482 (38.4) | 548 (38.4) | 519 (38.0) | 554 (40.6) |
| 75< | 317 (22.9) | 295 (23.2) | 341 (23.7) | 338 (22.8) | 251 (22.0) | 358 (28.5) | 320 (25.4) | 311 (27.7) | 313 (24.9) | 351 (24.6) | 349 (25.5) | 347 (25.4) |
| male (%) | 921 (66.4) | 808 (63.5) | 936 (65.1) | 969 (65.3) | 761 (66.8) | 835 (66.4) | 803 (63.6) | 730 (65.0) | 855 (68.1) | 906 (63.5) | 907 (66.3) | 885 (64.9) |
| BMI≥25 (%) | 366 (26.4) | 367 (28.9) | 376 (26.1) | 426 (28.7) | 284 (24.9) | 338 (26.9) | 308 (24.4) | 291 (25.9) | 299 (23.8) | 338 (23.7) | 344 (25.2) | 344 (25.2) |
| Preoperative chemotherapy (%) | 138 (9.9) | 109 (8.6) | 119 (8.3) | 112 (7.6) | 117 (10.3) | 127 (10.1) | 148 (11.7) | 123 (11.0) | 119 (9.5) | 129 (9.0) | 114 (8.3) | 91 (6.7) |
| DM (%) | 245 (17.7) | 243 (19.1) | 231 (16.1) | 243 (16.4) | 211 (18.5) | 243 (19.3) | 259 (20.5) | 210 (18.7) | 238 (19.0) | 253 (17.7) | 234 (17.1) | 273 (20.0) |
| Brinkman index (%) |  |  |  |  |  |  |  |  |  |  |  |  |
| 0 | 646 (46.6) | 636 (50.0) | 681 (47.4) | 735 (49.6) | 514 (45.1) | 624 (49.6) | 618 (49.0) | 533 (47.5) | 592 (47.2) | 697 (48.8) | 665 (48.6) | 658 (48.2) |
| <400 | 262 (18.9) | 210 (16.5) | 251 (17.5) | 247 (16.7) | 200 (17.5) | 210 (16.7) | 191 (15.1) | 186 (16.6) | 214 (17.1) | 253 (17.7) | 219 (16.0) | 250 (18.3) |
| ≥400 | 479 (34.5) | 426 (33.5) | 506 (35.2) | 501 (33.8) | 426 (37.4) | 424 (33.7) | 453 (35.9) | 404 (36.0) | 449 (35.8) | 477 (33.4) | 483 (35.3) | 456 (33.4) |
| Habitual alcohol intake (%) | 508 (36.6) | 440 (34.6) | 510 (35.5) | 504 (34.0) | 386 (33.9) | 423 (33.6) | 412 (32.6) | 395 (35.2) | 429 (34.2) | 479 (33.6) | 470 (34.4) | 480 (35.2) |
| COPD (%) | 43 (3.1) | 54 (4.2) | 50 (3.5) | 49 (3.3) | 42 (3.7) | 50 (4.0) | 39 (3.1) | 27 (2.4) | 48 (3.8) | 52 (3.6) | 37 (2.7) | 52 (3.8) |
| Congestive heart disease (%) | 6 (0.4) | 1 (0.1) | 5 (0.3) | 4 (0.3) | 5 (0.4) | 8 (0.6) | 6 (0.5) | 8 (0.7) | 5 (0.4) | 5 (0.4) | 6 (0.4) | 5 (0.4) |
| Ischemic heart disease (%) | 13 (0.9) | 9 (0.7) | 11 (0.8) | 16 (1.1) | 14 (1.2) | 17 (1.4) | 11 (0.9) | 13 (1.2) | 18 (1.4) | 7 (0.5) | 13 (1.0) | 17 (1.2) |
| Hypertension(%) | 567 (40.9) | 510 (40.1) | 561 (39.0) | 615 (41.5) | 468 (41.1) | 536 (42.6) | 533 (42.2) | 458 (40.8) | 506 (40.3) | 549 (38.5) | 562 (41.1) | 610 (44.7) |
| Dialysis (%) | 6 (0.4) | 10 (0.8) | 9 (0.6) | 6 (0.4) | 8 (0.7) | 3 (0.2) | 6 (0.5) | 7 (0.6) | 3 (0.2) | 3 (0.2) | 6 (0.4) | 5 (0.4) |
| Cerebrovascular disease (%) | 47 (3.4) | 40 (3.1) | 44 (3.1) | 74 (5.0) | 45 (3.9) | 59 (4.7) | 53 (4.2) | 50 (4.5) | 49 (3.9) | 51 (3.6) | 57 (4.2) | 50 (3.7) |
| Steroid (%) | 4 (0.3) | 9 (0.7) | 9 (0.6) | 15 (1.0) | 13 (1.1) | 15 (1.2) | 12 (1.0) | 10 (0.9) | 8 (0.6) | 13 (0.9) | 16 (1.2) | 7 (0.5) |
| Weight loss (%) | 20 (1.4) | 31 (2.4) | 25 (1.7) | 25 (1.7) | 30 (2.6) | 29 (2.3) | 27 (2.1) | 23 (2.0) | 22 (1.8) | 31 (2.2) | 22 (1.6) | 23 (1.7) |
| Bleeding disorder (%) | 127 (9.2) | 124 (9.7) | 129 (9.0) | 161 (10.9) | 121 (10.6) | 152 (12.1) | 143 (11.3) | 118 (10.5) | 123 (9.8) | 141 (9.9) | 136 (9.9) | 141 (10.3) |
| Preoperative transfusion (%) | 12 (0.9) | 12 (0.9) | 8 (0.6) | 13 (0.9) | 6 (0.5) | 9 (0.7) | 14 (1.1) | 15 (1.3) | 12 (1.0) | 11 (0.8) | 15 (1.1) | 16 (1.2) |
| ASA PS (%) |  |  |  |  |  |  |  |  |  |  |  |  |
| ASA1 | 274 (19.8) | 258 (20.3) | 263 (18.3) | 290 (19.6) | 207 (18.2) | 223 (17.7) | 226 (17.9) | 206 (18.3) | 243 (19.4) | 261 (18.3) | 239 (17.5) | 225 (16.5) |
| ASA2 | 964 (69.5) | 866 (68.1) | 1006 (70.0) | 1022 (68.9) | 804 (70.5) | 891 (70.8) | 877 (69.5) | 780 (69.5) | 876 (69.8) | 1019 (71.4) | 991 (72.5) | 984 (72.1) |
| ASA3_5 | 149 (10.7) | 148 (11.6) | 169 (11.8) | 171 (11.5) | 129 (11.3) | 144 (11.4) | 159 (12.6) | 137 (12.2) | 136 (10.8) | 147 (10.3) | 137 (10.0) | 155 (11.4) |
| T factor (%) |  |  |  |  |  |  |  |  |  |  |  |  |
| T0 | 12 (0.9) | 7 (0.6) | 9 (0.6) | 8 (0.5) | 8 (0.7) | 4 (0.3) | 4 (0.3) | 5 (0.4) | 4 (0.3) | 4 (0.3) | 5 (0.4) | 7 (0.5) |
| Tis | 19 (1.4) | 21 (1.7) | 16 (1.1) | 19 (1.3) | 18 (1.6) | 16 (1.3) | 18 (1.4) | 17 (1.5) | 17 (1.4) | 14 (1.0) | 14 (1.0) | 22 (1.6) |
| T1 | 260 (18.7) | 224 (17.6) | 260 (18.1) | 258 (17.4) | 219 (19.2) | 213 (16.9) | 178 (14.1) | 139 (12.4) | 151 (12.0) | 223 (15.6) | 210 (15.4) | 210 (15.4) |
| T2 | 277 (20.0) | 227 (17.8) | 267 (18.6) | 276 (18.6) | 206 (18.1) | 239 (19.0) | 224 (17.7) | 196 (17.5) | 215 (17.1) | 246 (17.2) | 272 (19.9) | 244 (17.9) |
| T3 | 637 (45.9) | 616 (48.4) | 681 (47.4) | 725 (48.9) | 533 (46.8) | 585 (46.5) | 647 (51.3) | 569 (50.7) | 664 (52.9) | 725 (50.8) | 650 (47.5) | 649 (47.6) |
| T4a | 142 (10.2) | 138 (10.8) | 168 (11.7) | 158 (10.7) | 129 (11.3) | 152 (12.1) | 147 (11.6) | 156 (13.9) | 157 (12.5) | 174 (12.2) | 167 (12.2) | 177 (13.0) |
| T4b | 40 (2.9) | 39 (3.1) | 37 (2.6) | 39 (2.6) | 27 (2.4) | 49 (3.9) | 44 (3.5) | 41 (3.7) | 47 (3.7) | 41 (2.9) | 49 (3.6) | 55 (4.0) |
| N factor (%) |  |  |  |  |  |  |  |  |  |  |  |  |
| N0 | 877 (63.2) | 771 (60.6) | 848 (59.0) | 878 (59.2) | 694 (60.9) | 760 (60.4) | 729 (57.8) | 614 (54.7) | 716 (57.1) | 799 (56.0) | 777 (56.8) | 786 (57.6) |
| N1a | 169 (12.2) | 181 (14.2) | 206 (14.3) | 223 (15.0) | 165 (14.5) | 145 (11.5) | 180 (14.3) | 158 (14.1) | 174 (13.9) | 210 (14.7) | 189 (13.8) | 157 (11.5) |
| N1b | 181 (13.0) | 165 (13.0) | 182 (12.7) | 177 (11.9) | 134 (11.8) | 165 (13.1) | 162 (12.8) | 175 (15.6) | 172 (13.7) | 194 (13.6) | 196 (14.3) | 200 (14.7) |
| N1c | 2 (0.1) | 1 (0.1) | 6 (0.4) | 3 (0.2) | 1 (0.1) | 1 (0.1) | 8 (0.6) | 2 (0.2) | 1 (0.1) | 2 (0.1) | 2 (0.1) | 1 (0.1) |
| N2a | 95 (6.8) | 98 (7.7) | 102 (7.1) | 122 (8.2) | 95 (8.3) | 100 (7.9) | 106 (8.4) | 102 (9.1) | 111 (8.8) | 138 (9.7) | 117 (8.6) | 123 (9.0) |
| N2b | 63 (4.5) | 56 (4.4) | 94 (6.5) | 80 (5.4) | 51 (4.5) | 87 (6.9) | 77 (6.1) | 72 (6.4) | 81 (6.5) | 84 (5.9) | 86 (6.3) | 97 (7.1) |
| M factor (%) |  |  |  |  |  |  |  |  |  |  |  |  |
| M0 | 1275 (91.9) | 1167 (91.7) | 1330 (92.5) | 1376 (92.8) | 1050 (92.1) | 1136 (90.3) | 1141 (90.4) | 1004 (89.4) | 1144 (91.2) | 1310 (91.8) | 1244 (91.0) | 1245 (91.3) |
| M1a | 94 (6.8) | 86 (6.8) | 82 (5.7) | 92 (6.2) | 69 (6.1) | 88 (7.0) | 88 (7.0) | 91 (8.1) | 86 (6.9) | 83 (5.8) | 93 (6.8) | 87 (6.4) |
| M1b | 12 (0.9) | 9 (0.7) | 19 (1.3) | 7 (0.5) | 16 (1.4) | 19 (1.5) | 22 (1.7) | 18 (1.6) | 18 (1.4) | 19 (1.3) | 15 (1.1) | 15 (1.1) |
| M1c | 6 (0.4) | 10 (0.8) | 7 (0.5) | 8 (0.5) | 5 (0.4) | 15 (1.2) | 11 (0.9) | 10 (0.9) | 7 (0.6) | 15 (1.1) | 15 (1.1) | 17 (1.2) |

| 2021 | 1 | 2 | 3 | 4 | 5 | 6 | 7 | 8 | 9 | 10 | 11 | 12 |
| --- | --- | --- | --- | --- | --- | --- | --- | --- | --- | --- | --- | --- |
| n | 1258 | 1221 | 1546 | 1513 | 1378 | 1538 | 1303 | 1309 | 1331 | 1382 | 1363 | 1408 |
| age (median [IQR]) | 69 [61, 75] | 69 [60, 75] | 69 [60, 75] | 69 [59, 76] | 70 [60, 76] | 69 [60, 75] | 70 [60, 76] | 69 [60, 76] | 69 [61, 74] | 70 [61, 77] | 69 [60, 75] | 69 [60, 76] |
| category of age (%) |  |  |  |  |  |  |  |  |  |  |  |  |
| <65 | 428 (34.0) | 414 (33.9) | 571 (36.9) | 563 (37.2) | 478 (34.7) | 570 (37.1) | 447 (34.3) | 443 (33.8) | 462 (34.7) | 471 (34.1) | 469 (34.4) | 495 (35.2) |
| 65-75 | 520 (41.3) | 511 (41.9) | 595 (38.5) | 570 (37.7) | 529 (38.4) | 594 (38.6) | 521 (40.0) | 525 (40.1) | 579 (43.5) | 524 (37.9) | 560 (41.1) | 560 (39.8) |
| 75< | 310 (24.6) | 296 (24.2) | 380 (24.6) | 380 (25.1) | 371 (26.9) | 374 (24.3) | 335 (25.7) | 341 (26.1) | 290 (21.8) | 387 (28.0) | 334 (24.5) | 353 (25.1) |
| male (%) | 829 (65.9) | 805 (65.9) | 969 (62.7) | 946 (62.5) | 873 (63.4) | 1020 (66.3) | 820 (62.9) | 849 (64.9) | 854 (64.2) | 914 (66.1) | 915 (67.1) | 907 (64.4) |
| BMI≥25 (%) | 345 (27.4) | 315 (25.8) | 383 (24.8) | 402 (26.6) | 377 (27.4) | 416 (27.0) | 343 (26.3) | 299 (22.8) | 335 (25.2) | 346 (25.0) | 329 (24.1) | 352 (25.0) |
| Preoperative chemotherapy (%) | 116 (9.2) | 100 (8.2) | 126 (8.2) | 115 (7.6) | 125 (9.1) | 149 (9.7) | 131 (10.1) | 140 (10.7) | 148 (11.1) | 127 (9.2) | 124 (9.1) | 121 (8.6) |
| DM (%) | 220 (17.5) | 202 (16.5) | 313 (20.2) | 280 (18.5) | 256 (18.6) | 288 (18.7) | 213 (16.3) | 255 (19.5) | 239 (18.0) | 257 (18.6) | 277 (20.3) | 264 (18.8) |
| Brinkman index (%) |  |  |  |  |  |  |  |  |  |  |  |  |
| 0 | 596 (47.4) | 589 (48.2) | 726 (47.0) | 716 (47.3) | 636 (46.2) | 720 (46.8) | 644 (49.4) | 638 (48.7) | 587 (44.1) | 671 (48.6) | 638 (46.8) | 636 (45.2) |
| <400 | 209 (16.6) | 186 (15.2) | 267 (17.3) | 271 (17.9) | 223 (16.2) | 262 (17.0) | 211 (16.2) | 198 (15.1) | 276 (20.7) | 241 (17.4) | 239 (17.5) | 298 (21.2) |
| ≥400 | 453 (36.0) | 446 (36.5) | 553 (35.8) | 526 (34.8) | 519 (37.7) | 556 (36.2) | 448 (34.4) | 473 (36.1) | 468 (35.2) | 470 (34.0) | 486 (35.7) | 474 (33.7) |
| Habitual alcohol intake (%) | 446 (35.5) | 404 (33.1) | 535 (34.6) | 533 (35.2) | 479 (34.8) | 541 (35.2) | 454 (34.8) | 443 (33.8) | 454 (34.1) | 482 (34.9) | 464 (34.0) | 504 (35.8) |
| COPD (%) | 46 (3.7) | 26 (2.1) | 50 (3.2) | 55 (3.6) | 48 (3.5) | 55 (3.6) | 44 (3.4) | 46 (3.5) | 31 (2.3) | 41 (3.0) | 46 (3.4) | 54 (3.8) |
| Congestive heart disease (%) | 9 (0.7) | 9 (0.7) | 13 (0.8) | 11 (0.7) | 3 (0.2) | 14 (0.9) | 5 (0.4) | 2 (0.2) | 7 (0.5) | 4 (0.3) | 5 (0.4) | 7 (0.5) |
| Ischemic heart disease (%) | 17 (1.4) | 12 (1.0) | 15 (1.0) | 19 (1.3) | 14 (1.0) | 18 (1.2) | 7 (0.5) | 14 (1.1) | 10 (0.8) | 10 (0.7) | 17 (1.2) | 15 (1.1) |
| Hypertension(%) | 549 (43.6) | 498 (40.8) | 663 (42.9) | 636 (42.0) | 556 (40.3) | 631 (41.0) | 560 (43.0) | 551 (42.1) | 569 (42.7) | 579 (41.9) | 573 (42.0) | 574 (40.8) |
| Dialysis (%) | 7 (0.6) | 6 (0.5) | 6 (0.4) | 7 (0.5) | 0 (0.0) | 9 (0.6) | 2 (0.2) | 7 (0.5) | 6 (0.5) | 4 (0.3) | 8 (0.6) | 5 (0.4) |
| Cerebrovascular disease (%) | 55 (4.4) | 47 (3.8) | 61 (3.9) | 56 (3.7) | 47 (3.4) | 60 (3.9) | 44 (3.4) | 59 (4.5) | 49 (3.7) | 66 (4.8) | 50 (3.7) | 41 (2.9) |
| Steroid (%) | 8 (0.6) | 11 (0.9) | 10 (0.6) | 12 (0.8) | 8 (0.6) | 12 (0.8) | 9 (0.7) | 12 (0.9) | 6 (0.5) | 15 (1.1) | 9 (0.7) | 11 (0.8) |
| Weight loss (%) | 24 (1.9) | 23 (1.9) | 36 (2.3) | 26 (1.7) | 34 (2.5) | 27 (1.8) | 31 (2.4) | 32 (2.4) | 37 (2.8) | 36 (2.6) | 26 (1.9) | 46 (3.3) |
| Bleeding disorder (%) | 136 (10.8) | 138 (11.3) | 174 (11.3) | 157 (10.4) | 142 (10.3) | 150 (9.8) | 144 (11.1) | 141 (10.8) | 143 (10.7) | 153 (11.1) | 143 (10.5) | 134 (9.5) |
| Preoperative transfusion (%) | 15 (1.2) | 9 (0.7) | 13 (0.8) | 18 (1.2) | 7 (0.5) | 16 (1.0) | 7 (0.5) | 7 (0.5) | 10 (0.8) | 7 (0.5) | 13 (1.0) | 14 (1.0) |
| ASA PS (%) |  |  |  |  |  |  |  |  |  |  |  |  |
| ASA1 | 234 (18.6) | 227 (18.6) | 254 (16.4) | 267 (17.6) | 230 (16.7) | 257 (16.7) | 203 (15.6) | 206 (15.7) | 214 (16.1) | 217 (15.7) | 220 (16.1) | 233 (16.5) |
| ASA2 | 879 (69.9) | 865 (70.8) | 1122 (72.6) | 1084 (71.6) | 994 (72.1) | 1105 (71.8) | 935 (71.8) | 953 (72.8) | 966 (72.6) | 1012 (73.2) | 989 (72.6) | 1038 (73.7) |
| ASA3_5 | 145 (11.5) | 129 (10.6) | 170 (11.0) | 162 (10.7) | 154 (11.2) | 176 (11.4) | 165 (12.7) | 150 (11.5) | 151 (11.3) | 153 (11.1) | 154 (11.3) | 137 (9.7) |
| T factor (%) |  |  |  |  |  |  |  |  |  |  |  |  |
| T0 | 6 (0.5) | 7 (0.6) | 13 (0.8) | 7 (0.5) | 6 (0.4) | 7 (0.5) | 10 (0.8) | 3 (0.2) | 6 (0.5) | 6 (0.4) | 7 (0.5) | 9 (0.6) |
| Tis | 20 (1.6) | 15 (1.2) | 21 (1.4) | 10 (0.7) | 21 (1.5) | 18 (1.2) | 20 (1.5) | 15 (1.1) | 16 (1.2) | 20 (1.4) | 14 (1.0) | 19 (1.3) |
| T1 | 227 (18.0) | 209 (17.1) | 274 (17.7) | 253 (16.7) | 241 (17.5) | 234 (15.2) | 209 (16.0) | 215 (16.4) | 206 (15.5) | 212 (15.3) | 206 (15.1) | 205 (14.6) |
| T2 | 257 (20.4) | 217 (17.8) | 277 (17.9) | 306 (20.2) | 239 (17.3) | 281 (18.3) | 222 (17.0) | 236 (18.0) | 246 (18.5) | 245 (17.7) | 239 (17.5) | 255 (18.1) |
| T3 | 569 (45.2) | 597 (48.9) | 746 (48.3) | 727 (48.1) | 668 (48.5) | 762 (49.5) | 651 (50.0) | 636 (48.6) | 658 (49.4) | 695 (50.3) | 682 (50.0) | 688 (48.9) |
| T4a | 139 (11.0) | 151 (12.4) | 171 (11.1) | 164 (10.8) | 153 (11.1) | 197 (12.8) | 157 (12.0) | 159 (12.1) | 156 (11.7) | 161 (11.6) | 169 (12.4) | 190 (13.5) |
| T4b | 40 (3.2) | 25 (2.0) | 44 (2.8) | 46 (3.0) | 50 (3.6) | 39 (2.5) | 34 (2.6) | 45 (3.4) | 43 (3.2) | 43 (3.1) | 46 (3.4) | 42 (3.0) |
| N factor (%) |  |  |  |  |  |  |  |  |  |  |  |  |
| N0 | 774 (61.5) | 728 (59.6) | 906 (58.6) | 913 (60.3) | 824 (59.8) | 892 (58.0) | 740 (56.8) | 771 (58.9) | 762 (57.3) | 794 (57.5) | 802 (58.8) | 821 (58.3) |
| N1a | 151 (12.0) | 178 (14.6) | 206 (13.3) | 200 (13.2) | 214 (15.5) | 210 (13.7) | 191 (14.7) | 172 (13.1) | 189 (14.2) | 189 (13.7) | 194 (14.2) | 194 (13.8) |
| N1b | 164 (13.0) | 137 (11.2) | 219 (14.2) | 180 (11.9) | 158 (11.5) | 220 (14.3) | 183 (14.0) | 180 (13.8) | 190 (14.3) | 195 (14.1) | 170 (12.5) | 186 (13.2) |
| N1c | 5 (0.4) | 4 (0.3) | 3 (0.2) | 7 (0.5) | 2 (0.1) | 5 (0.3) | 1 (0.1) | 1 (0.1) | 3 (0.2) | 3 (0.2) | 2 (0.1) | 3 (0.2) |
| N2a | 90 (7.2) | 99 (8.1) | 126 (8.2) | 130 (8.6) | 104 (7.5) | 118 (7.7) | 103 (7.9) | 106 (8.1) | 108 (8.1) | 111 (8.0) | 113 (8.3) | 136 (9.7) |
| N2b | 74 (5.9) | 75 (6.1) | 86 (5.6) | 83 (5.5) | 76 (5.5) | 93 (6.0) | 85 (6.5) | 79 (6.0) | 79 (5.9) | 90 (6.5) | 82 (6.0) | 68 (4.8) |
| M factor (%) |  |  |  |  |  |  |  |  |  |  |  |  |
| M0 | 1149 (91.3) | 1121 (91.8) | 1426 (92.2) | 1405 (92.9) | 1271 (92.2) | 1417 (92.1) | 1192 (91.5) | 1222 (93.4) | 1208 (90.8) | 1274 (92.2) | 1250 (91.7) | 1305 (92.7) |
| M1a | 79 (6.3) | 75 (6.1) | 98 (6.3) | 72 (4.8) | 71 (5.2) | 91 (5.9) | 86 (6.6) | 65 (5.0) | 88 (6.6) | 84 (6.1) | 77 (5.6) | 80 (5.7) |
| M1b | 20 (1.6) | 17 (1.4) | 16 (1.0) | 20 (1.3) | 20 (1.5) | 14 (0.9) | 13 (1.0) | 13 (1.0) | 22 (1.7) | 13 (0.9) | 21 (1.5) | 11 (0.8) |
| M1c | 10 (0.8) | 8 (0.7) | 6 (0.4) | 16 (1.1) | 16 (1.2) | 16 (1.0) | 12 (0.9) | 9 (0.7) | 13 (1.0) | 11 (0.8) | 15 (1.1) | 12 (0.9) |
